# Tailoring the visual communication of climate projections for local adaptation practitioners in Germany and the UK

**DOI:** 10.1098/rsta.2014.0457

**Published:** 2015-11-28

**Authors:** Susanne Lorenz, Suraje Dessai, Piers M. Forster, Jouni Paavola

**Affiliations:** School of Earth and Environment and ESRC Centre for Climate Change Economics and Policy, University of Leeds, Leeds LS2 9JT, UK

**Keywords:** climate change adaptation, climate projections, visualization, communication, decision-making, local government

## Abstract

Visualizations are widely used in the communication of climate projections. However, their effectiveness has rarely been assessed among their target audience. Given recent calls to increase the usability of climate information through the tailoring of climate projections, it is imperative to assess the effectiveness of different visualizations. This paper explores the complexities of tailoring through an online survey conducted with 162 local adaptation practitioners in Germany and the UK. The survey examined respondents’ assessed and perceived comprehension (PC) of visual representations of climate projections as well as preferences for using different visualizations in communicating and planning for a changing climate. Comprehension and use are tested using four different graph formats, which are split into two pairs. Within each pair the information content is the same but is visualized differently. We show that even within a fairly homogeneous user group, such as local adaptation practitioners, there are clear differences in respondents’ comprehension of and preference for visualizations. We do not find a consistent association between assessed comprehension and PC or use within the two pairs of visualizations that we analysed. There is, however, a clear link between PC and use of graph format. This suggests that respondents use what they think they understand the best, rather than what they actually understand the best. These findings highlight that audience-specific targeted communication may be more complex and challenging than previously recognized.

## 1. Introduction

Adaptation to climate change is inevitable [1]. Climate projections—‘simulated response[s] of the climate system to a scenario of future emission or concentration of greenhouse gases and aerosols’ [2]—are often used in scientific analysis and risk assessments to help decision-makers understand the risks posed by climate change and plan accordingly. This preparation for climate risks can also be described as planned adaptation to climate change, which is considered to be ‘the result of a deliberate policy decision, based on an awareness that conditions have changed or are about to change’ [3]. If maladaptation is to be avoided and decision-making made effective, then climate projections and information need to be usable by those people in the private and public sphere who have to practically prepare and plan for the impacts of a changing climate, namely adaptation practitioners. Borrowing from Lehmann *et al*. [4], we define adaptation practitioners as ‘decision-makers in the field of planned climate adaptation’. Specifically, in our research, we study adaptation practitioners within local government in Germany and the UK.

Climate projections are often communicated visually; the change of temperature over time for example is most often displayed in the form of a line graph, whereas bar charts are usually used to show precipitation amounts. With graphic representation of climate data being a key means of communicating these data, it is important to examine the usability of visualizations closely. Some research has already been conducted on the role of climate visualizations in the fields of climate change [5], impacts [6], modelling and projections [7], and adaptation and decision-making [8,9]. Moreover, lessons can also be learnt from research on visualization of risk and other information in the health and cognitive sciences [10–12], environmental hazards and geosciences [13–15], risk [16,17], design [18], computing [19,20] and hydrology [21,22]. Nevertheless, the lack of empirical work on visual communication is acknowledged and more research on visualization of uncertainty has been called for [13,14,23].

The existing literature suggests that visualizations and communication ought to support user needs [14] and be tailored to the target audience [23,24]. Tailoring has been suggested as one way to bridge the usability gap, i.e. the gap between the information produced by users and the information considered as usable by users [25]. Usability is understood as the combined ‘perception of usefulness and the actual capacity (…) to use different kinds of information’ [26]. The concept of tailoring of visualizations thus speaks to the understanding that different audiences have different perceptions, capacities and characteristics, which will impact their interpretation of a visualization [24]. Tailoring, therefore, aims to better understand these audience-specific aspects and customize or individualize visualizations accordingly to increase their effectiveness [27]. As to climate information, aspects that might be tailored specifically to audience needs could include, but are not limited to, the content of the visualization (e.g. showing mean temperature rise or showing maximum temperature rise), hue and saturation of colour [7], the inclusion of relevant past experiences for comparison (such as the mean temperature of the 2003 summer when talking about temperature projections) [28] or the type of graph format (such as using a thermometer to show temperature rise, rather than a line graph) [29].

For the effectiveness of visualizations to be increased, Stephens *et al*. [30] in their review of the communication of climate model ensembles, found that it is important to consider the balance between richness (the amount of data represented), robustness (the representation of scientific confidence and consensus) and saliency (the relevance of the information to user needs) in a visualization. It has been put forward that the more detailed assessment of such user needs, also termed as ‘strategic listening’, can be achieved with help from the decision-sciences [31].

Ultimately, a more scientific approach to the communication of science is called for [31], which necessitates more and better evaluated case study research, particularly focusing on both the preferences and the understanding of visualizations [23]. At the same time, it has been highlighted that, while understanding user preference is important, there is a need to ensure that choice of visualization based on preference alone does not lead to misunderstanding [21], but enables the user to make ‘better informed’ decisions. Consequently, Pappenberger *et al*. [21] call for more research on how varying both the information content and different graph formats impacts on user comprehension. Assessing user comprehension and preferences is a complex undertaking because of discrepancies between subjective and objective knowledge of an issue [32], both being influenced by a variety of different cognitive and attitudinal measures [32,33]. Being aware of the distinction between the different types of knowledge or comprehension may thus help to get a better understanding of the potential inconsistencies between preferences and comprehension, found in previous studies [10–12]. Consequently, an increased understanding of both user preferences and comprehension will support better tailoring of climate information, which ultimately will make this information more usable [25,34].

Considering these complexities, is it really feasible to produce tailored visual climate information in practice? This paper examines this question by conducting an empirical experiment with local adaptation practitioners in Germany and the UK on the usability of visualizations of climate projections. Local adaptation practitioners are an under-researched group of users of climate information [35,36], despite being recognized as playing an important role in addressing the challenges posed by climate change [37,38]. We explored local adaptation practitioners’ understanding of and preferences for different visualizations of climate projections. Our aim is not to find one ‘ideal’ visualization, but rather to highlight the complexities involved in tailoring and improving the usability of climate information.

## 2. Methodology

An online survey was developed to explore how local adaptation practitioners in Germany and the UK interpret visual representations (hereafter referred to as graph formats) of climate projections. The survey design, despite asking hypothetical questions, allowed us to collect empirical data that will nevertheless be reflective of decision and communication scenarios for adaptation practitioners. Both countries are considered to be among the leaders of climate change adaptation in Europe [39,40], but exhibit differences in terms of the extent to which adaptation has become a discrete policy field [41] and in terms of how scientific uncertainty is communicated in national adaptation strategies [42]. Owing to the context-specific nature of climate information for decision-making, tailoring and usability will have to be examined at a more local scale. Keeping in mind the national differences between the two countries, we explore differences and similarities in the comprehension of and preference for information provision at the local level that can help to inform the tailoring of climate information and its visualizations.

The aim of the survey was to better understand both participants’ comprehension of and their preferences for different graph formats in planning, decision-making and communicating adaptation in their organizations. We purposefully sampled employees in local government who work on environmental policy, climate change, sustainability or adaptation. Participants were recruited through direct email, advertisements in newsletters and Web portals, and through networks of relevant organizations such as the UK Climate Impacts Programme, the Local Government Association Climate Local Online Forum and the Klimaplattform. All participants completed the same questions and were not randomized. The survey was administered in German and English, and was translated by the lead researcher, to ensure consistency of the questions. Responses were collected from March to July 2013 in the UK (*n*=99) and from October 2013 to February 2014 in Germany (*n*=63). Individuals entering the survey were not offered any incentives or monetary rewards in return for their participation.

### (a) Development of different visualizations (graph formats)

**Figure 1.**
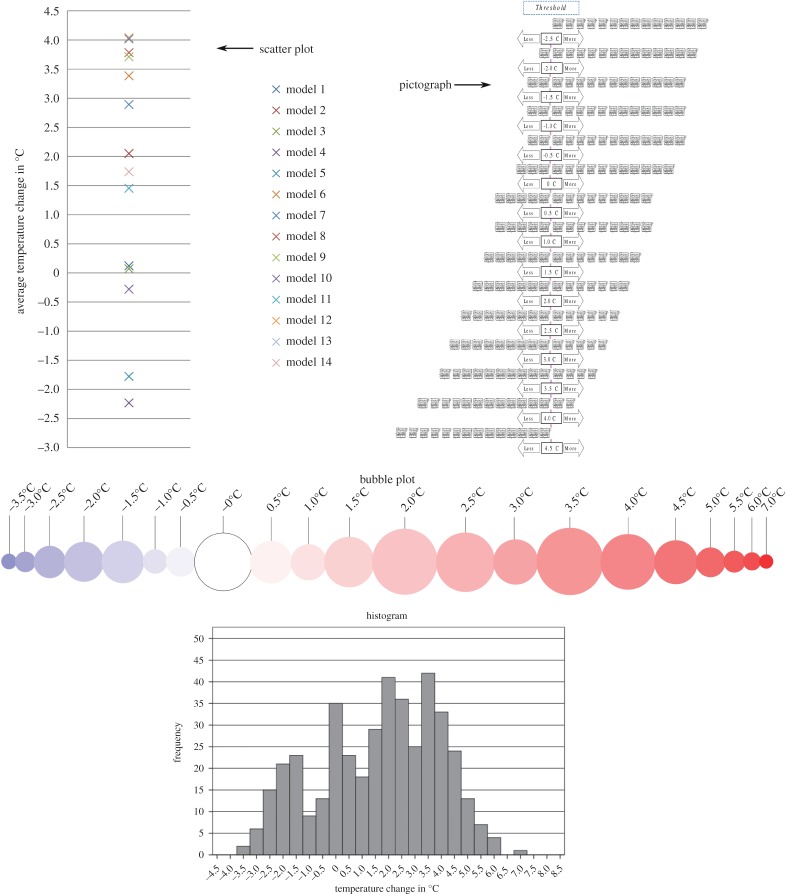
The four graph formats that were used in the survey. Each one of them also contained a figure caption explaining the data and the concept of thefigure. (Full-sized figures can be found in the electronic supplementary material.)

Four graph formats were developed to visualize the output of 14 General Circulation Models (GCMs) from the fifth phase of the Climate Model Intercomparison Project (CMIP5). The graph formats used in the two countries were based on output values for the grid cell around Newcastle, UK, in order to expose the participants from both countries to the same climate information. The choice of the grid cell is irrelevant for the experiment, as the purpose was only to extract data from the climate models for a given location. Of the four graph formats used (figure 1), two can be considered ‘traditional’ (linear scatter plot and histogram) and the other two ‘alternative’ (pictograph and bubble plot). We split these graph formats into two pairs, each containing one traditional and one alternative graph format showing the same information content within each pair, but with information content between pairs being different. Both pairs, however, used the same underlying data.

— **Pair 1:** The scatter plot and the pictograph show the change in mean summer temperature for the 2050s (2040–2069) under the Representative Concentration Pathway 6.0 [43], a medium greenhouse gas concentration trajectory, relative to a historical baseline period (1975–2004). The plots thus show 30 year seasonal mean changes for each of the 14 GCMs.
— **Pair 2:** The histogram and the bubble plot show the frequency for ranges of change in summer temperature for the 2050s (2040–2069) under the Representative Concentration Pathway 6.0 [43], a medium greenhouse gas concentration trajectory, relative to a historical baseline period (1975–2004). The plots are based on annual summer changes for each of the 30 years for each of the 14 GCMs.

### (b) Experimental procedure

To begin with, the survey participants were given a brief introduction to the survey and the aims of the research project, including information on confidentiality and informed consent. The climate data visualized in the survey were briefly explained and, although exactly the same data and graph formats were shown in both surveys, the English survey stated that the values were for a location in northeast England, whereas the German participants were informed that it was for a location in northeast Germany. This was done to ensure that the participants from both countries felt that the data shown would be relevant to their national contexts.

### (c) Criterion assessment

The aim of this analysis was to assess four key criteria within the two pairs: assessed and perceived comprehension (PC); use by self and use for showing to others, further explained below.

#### (i) Assessed and perceived comprehension

Respondents were shown the four graphs in the following order: (i) scatter plot, (ii) histogram, (iii) pictograph, and (iv) bubble plot. Respondents were not informed that the information content shown within pairs was the same and we deliberately showed the figures in this order so that respondents would alternate between pairs and the different information content and questions, so that practice effects could be kept to a minimum. Respondents were asked to answer the following multiple choice questions about the graph formats.

Pair 1: scatter plot and pictograph.
  — How many models project a decrease in summer temperature?
  — How many models project an increase in summer temperature by more than 3.0°C?
  — None of the models project a temperature change above which temperature value (to the nearest half of a degree)?
Pair 2: histogram and bubble plot.
  — Which is the most likely temperature change projected by the models?
  — What is the range of projected temperature change in the figure?
  — Which value is more likely, −2.5°C or 5.0°C?
  — Are you more likely to get a temperature change below −2.5°C or above 5.0°C?

Every response was coded ‘0’ for incorrect and ‘1’ for correct answers. An assessed comprehension score (ACS) was created by calculating the mean of the coded answers for each respondent for each figure. To assess perceived comprehension (PC), respondents were asked ‘Which figure did you find the easiest to understand?’, with the option of choosing any one of the four formats.

#### (ii) Use by self and use for showing to others

Local adaptation practitioners not only consume climate information for their own use and planning, but also communicate it further to colleagues, managers or elected representatives. Therefore, we assessed the preferences for the use of graph formats that is both inward-facing (use by self) and outward-facing (use for showing to others). Use by self relates to individual decision-making. Preferences and perceived usability of graph formats for use by self were assessed by asking ‘If you had to make a planning decision, which of these figures would you find most helpful for your decision-making process?’ Respondents could choose one of the four graph formats or ‘Depends on the decision’ or ‘None of the above’. Preferences for use for showing to others were assessed by the question ‘If you had to persuade someone in your organization (e.g. your colleagues or your boss) of the necessity to start planning for changes in future summer temperatures, which one of these figures would you choose?’ Respondents could choose one of the four graph formats or ‘I wouldn’t use a figure at all’. Perceived comprehension, use by self and use for showing to others were recoded into a binary variable (1=selected, 0=not selected) for each of the four graph types. These binary variables were subsequently used in the Spearman’s rank order correlation tests described in §3b. The survey also collected qualitative data, as respondents had the opportunity to leave further explanations of their choices in comments boxes for the questions on perceived comprehension, use by self and use for showing to others.

### (d) Other sample characteristics and sample description

Table 1 gives an overview of the other sample characteristics for the two samples. The UK sample is somewhat younger than the German sample and thus has a higher percentage of respondents with fewer years of relevant work experience, but in the main the two samples are comparable.

**Table 1.** Sample description.

	UK sample (*n*=99) (%)	German sample (*n*=63) (%)
gender		
female	40.4	42.9
male	59.6	57.1
age groups		
20–29 years	13.1	3.2
30–39 years	36.4	22.2
40–49 years	30.3	27.0
50–59 years	16.2	39.7
60 and over	4.0	7.9
education		
degree or higher academic qualification	92.9	100
no degree or higher academic qualification	7.1	
work experience in a related job		
0–5 years	17.2	15.9
6–10 years	32.3	17.5
11–15 years	20.2	14.3
16–20 years	9.1	3.2
21–25 years	7.1	25.4
26–30 years	5.1	15.9
31–35 years	4.0	4.8
36–40 years	5.1	3.2
colour blind	2	0

Three measures around self-assessed knowledge and experience were included: (i) level of engagement with climate projections (‘How much do you engage with climate projections in your day-to-day job?), (ii) involvement in adaptation in work within the organization (‘Have you been actively involved in the climate change adaptation process in your organization?’), and (iii) climate change knowledge (‘How good is your knowledge of the topic of climate change?’). These three measures were assessed on a 6-point Likert scale with 1 being the ‘least favourable’ and 6 being the ‘most favourable’ option. As the survey also collected data using the subjective numeracy scale developed by Fagerlin *et al*. [44], which measures individual scale items on a 6-point Likert scale, it was decided to use the same scale for all of the measures in the survey to ensure consistency.

We did not find any systematic effects of socio-demographics, self-assessed knowledge and experience or subjective numeracy on comprehension or use that were consistent across both country samples. Further details on these results can be found in the electronic supplementary material, S3.

## 3. Results

Following the production of descriptive statistics for the four key criteria and the other sample characteristics, it was decided to use non-parametric statistical analysis as the ACSs for the graph formats were not normally distributed [45].

### (a) Outcome description

**Figure 2.**
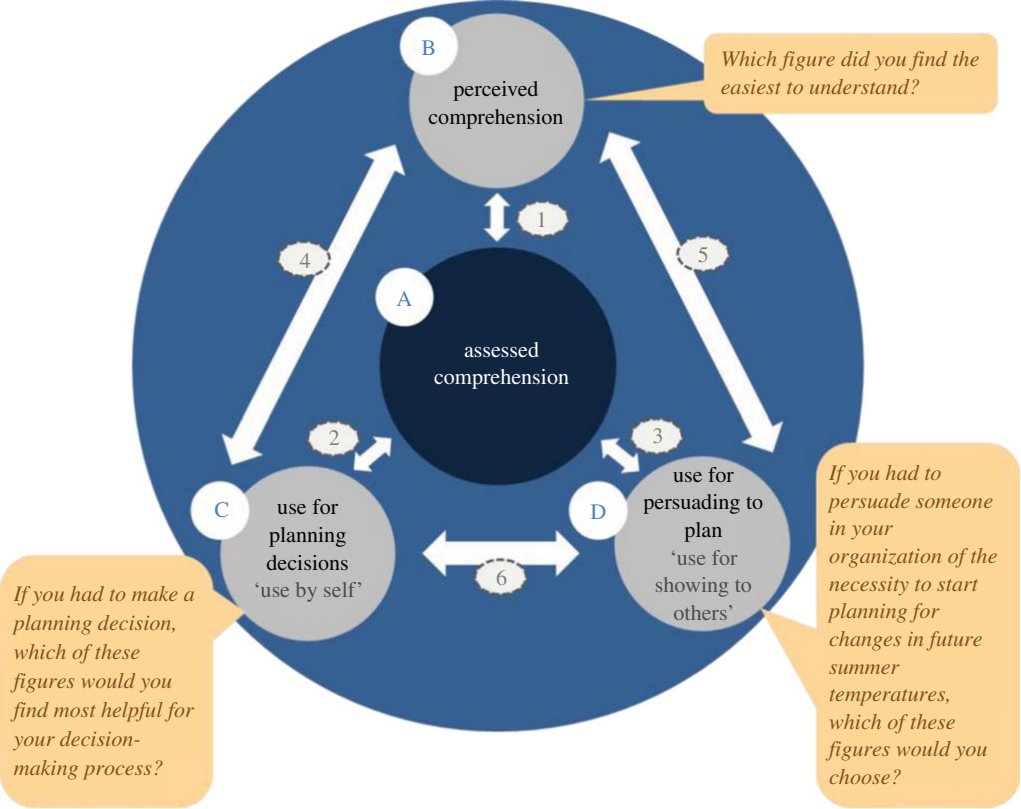
The four key criteria are denoted by capital letters: assessed comprehension (A); perceived comprehension (B); use for planning decisions—use by self (C); and use for persuading to plan—use for showing to others (D). The associations are represented with the numbered arrows (1–6).

We hypothesised at the outset that the four key criteria would be associated with each other. Figure 2 illustrates these hypothesized associations between assessed (A) and perceived comprehension (B) and use by self (C) and use for showing to others (D). In the following sections, we assess each criterion separately, followed by the relationships between them.

#### (i) Assessed comprehension (A)

Table 2 summarizes the mean ACS and standard deviation for each graph format in the two countries, as well as comparisons of the two samples. While the UK respondents achieved statistically significantly higher ACSs on the scatter plot, histogram and bubble plot than German respondents, they achieve a significantly lower ACS on the pictograph. Such a marked difference between assessed comprehension of the two samples for each of the four graph formats is interesting to note, especially given the similarity of the two country samples highlighted in table 1.

**Table 2.** ACSs for all graph formats. For the mean ACS higher values reflect better comprehension of the graph format; ACS was compared between countries with the Mann–Whitney *U* test, with entries in the three columns headed *U*, *z* and *r* providing the detailed test statistics.

	UK			Germany			ACS compared across both countries		
	mean	s.d.	median	mean	s.d.	median	*U*	*z*	*r*
pair 1									
scatter plot	0.88	0.17	1	0.70	0.23	0.67	***1761	−5.23	0.41
pictograph	0.49	0.29	0.33	0.62	0.32	0.67	**2391	−2.63	0.21
pair 2									
histogram	0.90	0.16	1	0.79	0.24	0.75	**2298	−3.21	0.25
bubble plot	0.88	0.15	1	0.80	0.22	0.75	*2494.5	−2.39	0.19

*p<0.05, **p<0.01 and ***p<0.001.

Examining the ACSs within each pair of visualizations in more detail using the Wilcoxon signed-rank test, we note a statistically significant drop of the ACS in pair 1 by 0.39 from the scatter plot to the pictograph in the UK sample (*z*=−7.36, *p*<0.0001, *r*=0.52). This is 0.31 more than in the German sample, where the ACSs on both the scatter plot and the pictograph do not differ significantly. In the second pair, the ACSs on both the histogram and the bubble plot do not differ significantly in either sample. We thus note that, within both pairs in the German sample, graph format did not affect assessed comprehension. Interestingly for the UK sample, this only holds true for pair 2 but not for pair 1, where the pictograph’s low ACS is noteworthy. This could be explained by ‘bad design’ affecting respondents’ ACS. In a study by Daron *et al*. [46], it was found that a similar graph format to the pictograph using the exceedance of thresholds was also the least preferred by respondents. However, this may only be a partial explanation, as we do not observe the same significant difference across both country samples. It may thus be that respondents in the UK might have been less willing to engage with something new or different, and therefore may have spent less time on trying to understand the graph format resulting in a lower ACS. The findings suggest that showing respondents different graph formats might not make much of a difference, unless the graph formats widely differ from what respondents are used to. In that case, assessed comprehension seems to be lower.

#### (ii) Perceived comprehension (B), use by self (C) and use for showing to others (D)

When examining the relationship between the original uncoded variables with the *χ*^2^-test for independence, we do not find any statistically significant difference between the UK and German respondents in PC (*χ*^2^(3,*n*=162)=4.08, *p*=0.25, Cramer’s *V* =0.16), use by self (*χ*^2^ (5, *n*=162)=8.59, *p*=0.13, Cramer’s *V* =0.23) or use for showing to others (*χ*^2^(4,*n*=162)=2.51, *p*=0.64, Cramer’s *V* =0.13). Respondents’ dichotomized choices of graph formats (selected or not selected) for all three variables have been summarized in the first data column in table 3. The qualitative explanations given by the respondents suggest that the three key reasons for the popularity of the histogram, in order of popularity, are: familiarity with the graph format, perceived clarity of display (also found to be important in Daron *et al*. [46]) and perceived ease of readability of frequencies. Some of this preference for the histogram may also be explained by the ‘frequency format hypothesis’, which stipulates that humans have evolved to find frequency distributions naturally easier to interpret [47]. However, not only has the explanatory power of this hypothesis been recently questioned [48], but we would also like to highlight that it may be that respondents simply perceived the other graph formats as less effective than the histogram due to their design. For use for showing to others the bubble plot is the second most popular format. Its higher ranking for use for showing to others compared with use by self could be explained by the view of local adaptation practitioners that they have to do some persuading and convincing to increase buy-in for adaptation actions. Qualitative survey responses suggest that the bubble plot is considered to be more visually persuasive and a good ‘initial hook’ for discussions.

**Table 3.** Correlations of ACS for each graph type across PC, use by self and use for showing to others. The percentage of respondents choosing the respective graph type for each of the criteria (PC, use by self and use for showing to others) is given in the first data column. The strength of the relationship between whether the respondents selected (‘yes’) or did not select (‘no’) the respective figure is then expressed through the Spearman correlation coefficient rho. S, scatter plot; P, pictograph; H, histogram; B, bubble plot; N/A, cannot be computed as the pictograph was not chosen by any respondent for use by self.

				UK					Germany				
					pair 1		pair 2			pair 1		pair 2	
				choice	S	P	H	B	choice	S	P	H	B
PC—ACS													
PC (1)	pair 1	S	yes	21.2	0.11				34.9	−0.07			
			no	78.8					65.1				
		P	yes	6.1		0.17			3.2		−0.03		
			no	93.9					96.8				
	pair 2	H	yes	**54**.**5**^a^			0.09		**47**.**6**^a^			−0.07	
			no	45.5					52.4				
		B	yes	18.2				0.02	14.3				0.11
			no	81.8					85.7				

use by self—ACS													
use by self (2)	pair 1	S	yes	13.1	−0.10				17.5	0.05			
			no	86.9					82.5				
		P	yes	5.1		0.19			0		N/A		
			no	94.9					100				
	pair 2	H	yes	**52**.**5**^a^			0.10		**42**.**9**^a^			−**0**.26*	
			no	47.5					57.1				
		B	yes	3				−0.08	11.1				0.02
			no	97					88.9				

use for showing to others—use by self													
use for showing to others (3)	pair 1	S	yes	9.1	−0.07				11.1	−0.13			
			no	90.9					88.9				
		P	yes	2		0.11			3.2		−0.09		
			no	98					96.8				
	pair 2	H	yes	**48**.**5**^a^			0.16		**52**.**4**^a^			−0.02	
			no	51.5					47.6				
		B	yes	24.2				0.15	25.4				0.10
			no	75.8					74.6				

^a^Most preferred graph format.

*p<0.05.

### (b) Differences in assessed comprehension across perceived comprehension and use (1, 2 and 3)

Having provided a brief overview of the four criteria, the following analysis will focus on the extent of association between these criteria. We conducted Spearman’s rank order correlation tests to examine the strength of the association between the ACS on each of the graph formats and respondents’ preferences to select or not select the respective graph format for perceived comprehension (1), use by self (2) or use for showing to others (3). The results of the tests are summarized in table 3.

We note that there is no consistent association between ACS and the other criteria for the graph formats within either pair. Only one of the associations of the 23 tested is statistically significant (*p*=0.04), but, as this association has been observed in isolation, it should be treated with caution due to the potential risk of a type I error in this case. The fact that we did not find consistent associations is interesting, given our initial hypothesis that the ACS would be associated with the other criteria. If respondents were better judges of their actual understanding of a graph format, we would have expected this to be at least reflected in higher correlation coefficients and more significant associations for the relationship between assessed and perceived comprehension. It is possible that other factors influence the association between assessed comprehension and use, such as the type of planning decision at hand or the prior knowledge and experience of the respective colleague(s) in question for use for showing to others. These factors may guide choice more than just assessed comprehension, but are more difficult to capture due to varying decision and communication contexts. We will return to this question in more detail in the discussion.

### (c) Relationship between perceived comprehension, use by self and use for showing to others (4, 5 and 6)

Our investigation into the relationship between PC, use by self and use for showing to others found a consistently strong link between each of them in both the UK and the German samples; see table 4 for details.

**Table 4.** Relationship between PC, use by self and use for showing to others. Entries are the Pearson’s *χ*^2^-values.

	*χ*^2^
PC—use by self	
UK	94.31***
Germany	46.74***
PC—use for showing to others	
UK	51.73***
Germany	37.37***
use by self—use for showing to others	
UK	68.89***
Germany	39.65**

**p<0.01 and ***p<0.001.

Furthermore, we note that, in the German sample, for the scatter plot, the histogram and the bubble plot the majority of the respective respondents picked the same figures both as easiest to understand (PC) and as appropriate for use by self. In the UK sample, we observed the same for the histogram and the pictograph; however, the majority of those who picked the scatter plot as easiest to understand (PC) would still pick the histogram for planning (use by self). In both samples, we found that the majority of respondents who picked the histogram or the bubble plot as the easiest to understand (PC) also picked it as the most persuasive when showing it to someone else. On the other hand, many of those who chose the scatter plot as the easiest to understand (PC) still picked the histogram for persuasion (use for showing to others). Lastly, we found that respondents’ choice of graph formats for use by self and use for showing to others was consistent. For this we see the strongest link for the histogram and the bubble plot in both samples.

What these results point towards is that, while perceived comprehension and use are strongly associated and respondents’ preferences are thus consistent, the lack of association of the three preference measures with assessed comprehension across both pairs appears to separate respondents’ subjective preferences from actual comprehension. This seems to indicate that respondents tend to use what they think they understand best, rather than what they actually understand best.

## 4. Discussion

**Figure 3.**
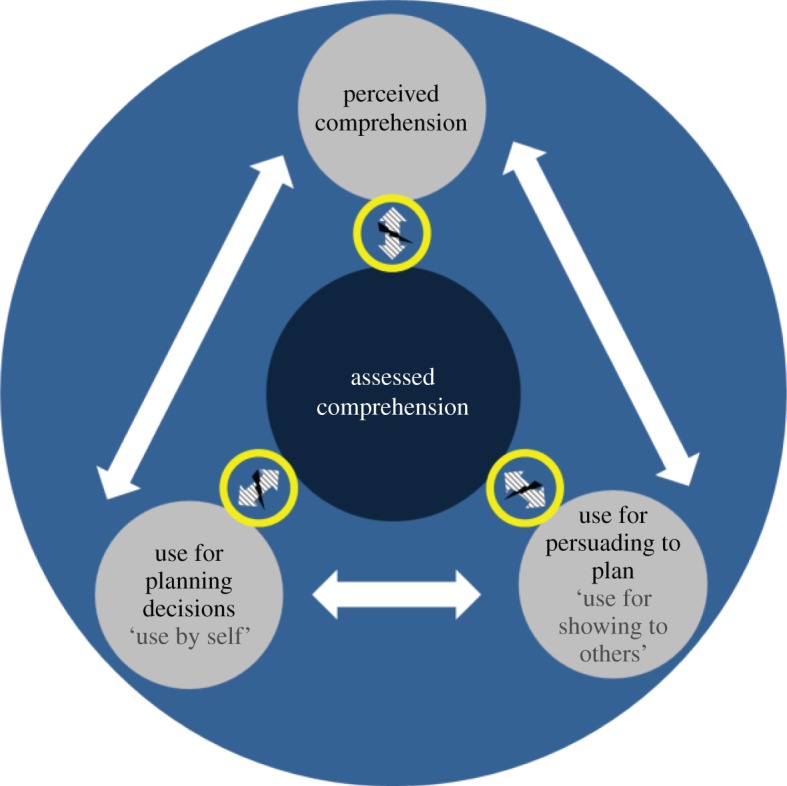
Associations between the four key criteria showing, on the one hand, the disconnect between users’ assessed comprehension and the other three key criteria, and, on the other hand, the strong relationship between perceived comprehension and use by self and use for showing to others.

The aim of this paper was to explore empirically the differences and similarities in the comprehension of and preference for different forms of visualization among adaptation practitioners in the UK and Germany. Our findings within both pairs of graph formats suggest that in both countries there is a disconnect between users’ assessed comprehension and subjective preference. However, there is a strong link between people’s perceived comprehension and their preferences for graph formats they use themselves and for communicating with colleagues and superiors about the necessity to take action on adaptation (figure 3). As we have observed the same associations and lack thereof across both pairs of graph formats, showing different information content, these observations seem to suggest that this is likely to be an issue encountered with visual communication of climate information more widely.

Our findings regarding the gap between comprehension and preference resonate with the results reported in the health sciences literature. Parrott *et al*. [11] found that people’s reading of familiar graph formats is affected by learned heuristics: respondents’ familiarity plays a bigger role in the process of reading and sense-making of graphs than the actual comprehension of the information shown. They argue that this could lead to a disconnect not only between the encoded and decoded meaning of the graph but also in respondents stating preferences for graphs that they do not understand as well as other graphs [11]. Our results also resonate with findings of a study of physicians’ assessment of visually displayed information, in which respondents’ preferences for graph formats and displays appeared to be based on familiarity with the graph formats rather than on their comprehension [12]. Qualitative explanations in our surveys also suggested this. The disconnect supports Ancker *et al*.’s [10] argument that, although it is important to focus on the preferences of information recipients, this may result in poor quantitative judgements. There is a complex interplay between respondents’ comprehension and preferences for use of visualizations in practice, and cognitive biases are involved in it. We need to be aware of them and consider how they could be dealt with or overcome if we are to make visual communication of climate projections more effective.

We note that the biases in information provision and use are consistent across the two samples. This is interesting considering the differences in relation to adaptation at the national level between the two countries [41,42]. This is not to say that local adaptation practitioners are a homogeneous group and that advice for tailoring is generalizable. On the contrary, the findings highlight that comprehension and preferences, and thus usability, are specific to the individual and in many cases likely to be connected to the stage of adaptation planning in a given local authority or municipality. Respondents highlighted that certain graph formats are better for initial persuasion needed to ensure buy-in into adaptation, whereas other formats communicate better the exact figures needed for more specific adaptive measures. The consistent cognitive biases and the within-group differences demonstrate that the demands for more ‘audience-specific communication’ may be more complex and challenging than has been recognized to date. To address these challenges, we make a number of suggestions based on the insights from our research.

Firstly, our results ought to be situated within the wider judgement and decision-making literature. Insights from this research have shown that, although often there are differences between self-reported and actual knowledge of climate change [32], they affect both people’s concern and risk perception of the topic [49–51]. Despite ‘knowledge of climate change’ being a broader construct than comprehension and preference for graph formats, which has been assessed in this study, we would nevertheless suggest that these systematic deviations of human judgement affecting the decision-making process pose similar challenges for climate visualizations. A better understanding of the cognitive factors influencing subjective and objective knowledge/comprehension may thus help to tailor visualizations more effectively. Moreover, while the literature has already called for a greater integration of the decision-sciences into the development of technical information [52] and into the wider question on communicating climate change [53], we suggest this integration needs to be made explicit also for the issue of visualizations. Just as much as visual material should not be considered as a simple add-on to the science communication process [31], use and comprehension of visualizations and their impact on communication and decision-making deserve more attention from the judgement and decision-making literature other than just as a subsection of the ‘climate change knowledge’ issue.

Secondly, some audiences are more likely to be susceptible to the change of graph format than others and thus innovative designs may not work equally well in different contexts. In the UK, we noted a significant drop in ACS from the scatter plot to the pictograph, which was not seen in the German sample. Additionally, we even noted a slight (although insignificant) increase in ACS from the histogram to the bubble plot in the latter sample. The role of familiarity, the willingness to engage with and the impact of new designs may thus be dependent on the audience. A better understanding of this may help to decide where best to target innovative visualizations and where it is better to use ‘tried and tested’ designs.

Lastly, based on the finding that some graph formats are considered to be more persuasive than others and thus may lend themselves more to certain communication aims, we suggest that more research should be done on understanding how to match visualizations with communication aims. Climate visualization, like science communication more widely, would benefit here from a much more interdisciplinary approach [31,54]. If designs were created collaboratively, based on more detailed knowledge of the cognitive comprehension and biases of the target audience, more persuasive and engaging, yet scientifically robust, visualizations could be created. Some of the concerns of climate scientists arise out of the worry that making something ‘easier to understand’ comes at the cost of scientific rigour [54], and we suggest that this concern can be overcome through joint design of visualizations.

In all of these suggestions, we see that what the field of information tailoring needs first and foremost is greater collaboration between different fields of expertise and between producers and users of information and we should thus consider co-design [55] alongside co-production. Lemos & Rood’s [56] argument that producers and users of knowledge have different assumptions as to what is useful and what is actually usable information should be applied also to the visual aspects of information provision. While research strives to find new and more effective ways of communication and visualization of information and impacts, we acknowledge that what is effective cannot necessarily be judged *a priori* by the information producers [6] without empirical testing. Even if individual mismatches between comprehension and preferences could be overcome or addressed, past research highlights that there are further cognitive challenges, such as confirmation bias, anchoring or belief persistence [57], and institutional complexities, such as different approaches to risk governance [58], that need to be considered in tailoring efforts. What is designed as the best fit for comprehension and preferences may not fit with the local institutional contexts and guidelines.

Throughout all of this, we cannot lose sight of the ulterior motive of climate science communication to foster action on adaptation and improve adaptive capacity. Strengthening adaptive capacity will often occur through social and organizational learning [59,60]. Vulturius & Swartling [60] found that learning and engagement with adaptation improved when information users could relate communicated scientific knowledge better to their contexts and needs, highlighting a need for more tailored information. If co-production and co-design of information were thus to take place alongside each other, it can be anticipated that learning is further increased also with an ultimate positive impact on adaptive capacity.

We acknowledge that there are potential limitations to our findings, such as self-selection bias: our sample may have more respondents with an inherent interest in visualization and under-represent the less interested. Owing to different computer display sizes and resolutions, some respondents reported not being able to see the entire visualization without scrolling, which may have affected their responses. However, self-selection bias is an issue that social science surveys will always have to be mindful of and seeing the visualizations did not appear to have been systematically problematic. Therefore, we do not think that these issues significantly impact our findings. Furthermore, it could also be that those who are less motivated to use climate projections may be less motivated to use formats that they perceive to be less easy to use (even if they are better at using them), which could impact on the relationship between assessed and perceived comprehension. Lastly, our statistical tests may have lower statistical power than ideally desirable because of the small sample size. Nevertheless, we have uncovered interesting patterns that are consistent across both samples, increasing our confidence in our findings. Further experimental data collection with larger samples and in more countries would allow for more rigorous statistical testing.

## 5. Conclusion

In the introduction, we highlighted that visualization of information faces the demands for more audience-specific tailoring, greater evaluation of its effectiveness and more empirical evidence. Yet, requests for the communication and visualization of climate change adaptation information to be more effective and understandable [34] and suggestions for the tailoring of climate information [25] have remained mostly within the theoretical realm. We report empirical evidence about the complexities involved in the visualization of information and tailoring of communication in practice. Our results highlight that ideal solutions for tailored communication of climate data for decision-making on adaptation may not be found and that their search may be problematic and futile because of a lack of within-group homogeneity and the disconnect between assessed and perceived comprehension and preferences for the use of graph formats. This does not mean that further advances in this field are not needed—our results just highlight that claims regarding effective visualizations need to be tested and verified with more veracity, as much within groups as between them.

We recognize that visual information provision to decision-makers is only a small part of the much more extensive process of co-production of knowledge and the facilitation of user–producer interaction. Yet visual information is a crucial issue if we are to consider the information provision and knowledge production process holistically. Our paper responded to the request for more empirical evidence, researching both adaptation practitioners’ comprehension and their preference for different visual formats for the communication of climate projections. We did not set out to find an ‘ideal’ visualization, but instead our results demonstrate that we need to invest more thought into how tailoring can be facilitated at the same time as realizing that, even though there may be no such thing as a universal solution to the tailoring question, co-design and increased empirical testing may take us some way towards more rather than most effective visualizations.

## Supplementary Material

Questionnaire - UK

## Supplementary Material

Questionnaire - Germany

## Supplementary Material

Effects of other sample characteristics on comprehension and use

## Supplementary Material

Survey data Germany

## Supplementary Material

Survey data UK

## Data accessibility

The original surveys distributed in English and in German have been uploaded as part of the electronic supplementary material, as well as the data collected in the surveys.

## References

[1] Moss RH *et al* 2013 Hell and high water: practice-relevant adaptation science. Science 342, 696–698. (10.1126/science.1239569) 24202163

[2] Planton S 2013 Annex III: glossary. In *Climate change 2013: the physical science basis. Contribution of Working Group I to the Fifth Assessment Report of the Intergovernmental Panel on Climate Change* (eds TF Stocker *et al.*). Cambridge, UK: Cambridge University Press.

[3] Fussel HM 2007 Adaptation planning for climate change: concepts, assessment approaches, and key lessons. Sustain. Sci. 2, 265–275. (10.1007/s11625-007-0032-y)

[4] Lehmann P, Brenck M, Gebhardt O, Schaller S, Sussbauer E 2015 Barriers and opportunities for urban adaptation planning: analytical framework and evidence from cities in Latin America and Germany. Mitig. Adapt. Strateg. Glob. Change 20, 75–97. (10.1007/s11027-013-9480-0)

[5] van der Linden S, Leiserowitz A, Feinberg G, Maibach E 2014 How to communicate the scientific consensus on climate change: plain facts, pie charts or metaphors? Clim. Change 126, 255–262. (10.1007/s10584-014-1190-4)

[6] MacLeod DA, Morse AP 2014 Visualizing the uncertainty in the relationship between seasonal average climate and malaria risk. Sci. Rep. 4, 7264 (10.1038/srep07264) 25449318 PMC4250912

[7] Kaye NR, Hartley A, Hemming D 2012 Mapping the climate: guidance on appropriate techniques to map climate variables and their uncertainty. Geosci. Model Dev. 5, 245–256. (10.5194/gmd-5-245-2012)

[8] Wong-Parodi G, Fischhoff B, Strauss B 2014 A method to evaluate the usability of interactive climate change impact decision aids. Clim. Change 126, 485–493. (10.1007/s10584-014-1226-9)

[9] Sheppard SRJ, Shaw A, Flanders D, Burch S, Wiek A, Carmichael J, Robinson J, Cohen S 2011 Future visioning of local climate change: a framework for community engagement and planning with scenarios and visualisation. Futures 43, 400–412. (10.1016/j.futures.2011.01.009)

[10] Ancker JS, Senathirajah Y, Kukafka R, Starren JB 2006 Design features of graphs in health risk communication: a systematic review. J. Am. Med. Inform. Assoc. 13, 608–618. (10.1197/jamia.M2115) 16929039 PMC1656964

[11] Parrott R, Silk K, Dorgan K, Condit C, Harris T 2005 Risk comprehension and judgments of statistical evidentiary appeals. Hum. Commun. Res. 31, 423–452. (10.1093/hcr/31.3.423)

[12] Elting LS, Martin CG, Cantor SB, Rubenstein EB 1999 Influence of data display formats on physician investigators’ decisions to stop clinical trials: prospective trial with repeated measures. Br. Med. J. 318, 1527–1531. (10.1136/bmj.318.7197.1527) 10356010 PMC27896

[13] Broad K, Leiserowitz A, Weinkle J, Steketee M 2007 Misinterpretations of the ‘cone of uncertainty’ in Florida during the 2004 hurricane season. Bull. Am. Meteorol. Soc. 88, 651–667. (10.1175/BAMS-88-5-651)

[14] Bostrom A, Anselin L, Farris J 2008 Visualizing seismic risk and uncertainty. Ann. N. Y. Acad. Sci. 1128, 29–40. (10.1196/annals.1399.005) 18469212

[15] Gahegan M 1999 Four barriers to the development of effective exploratory visualisation tools for the geosciences. Int. J. Geogr. Inf. Sci. 13, 289–309. (10.1080/136588199241210)

[16] Ibrekk H, Morgan MG 1987 Graphical communication of uncertain quantities to nontechnical people. Risk Anal. 7, 519–529. (10.1111/j.1539-6924.1987.tb00488.x)

[17] Hess R, Visschers VHM, Siegrist M 2011 Risk communication with pictographs: the role of numeracy and graph processing. Judgment Decis. Mak. 6, 263–274.

[18] Quispel A, Maes A 2014 Would you prefer pie or cupcakes? Preferences for data visualization designs of professionals and laypeople in graphic design. J. Vis. Lang. Comput. 25, 107–116. (10.1016/j.jvlc.2013.11.007)

[19] Kelleher C, Wagener T 2011 Ten guidelines for effective data visualization in scientific publications. Environ. Model. Softw. 26, 822–827. (10.1016/j.envsoft.2010.12.006)

[20] Sanyal J, Song Z, Bhattacharya G, Amburn P, Moorhead R 2009 A user study to compare four uncertainty visualization methods for 1D and 2D datasets. IEEE Trans. Vis. Comput. Graph. 15, 1209–1218. (10.1109/tvcg.2009.114) 19834191

[21] Pappenberger F, Stephens E, Thielen J, Salamon P, Demeritt D, Andel SJ, Wetterhall F, Alfieri L 2013 Visualizing probabilistic flood forecast information: expert preferences and perceptions of best practice in uncertainty communication. Hydrol. Process. 27, 132–146. (10.1002/hyp.9253)

[22] Gimesi L 2009 Development of a visualization method suitable to present tendencies of changes in precipitation. J. Hydrol. 377, 185–190. (10.1016/j.jhydrol.2009.08.027)

[23] Spiegelhalter D, Pearson M, Short I 2011 Visualizing uncertainty about the future. Science 333, 1393–1400. (10.1126/science.1191181) 21903802

[24] Nicholson-Cole SA 2005 Representing climate change futures: a critique on the use of images for visual communication. Comput. Environ. Urban Syst. 29, 255–273. (10.1016/j.compenvurbsys.2004.05.002)

[25] Lemos MC, Kirchhoff CJ, Ramprasad V 2012 Narrowing the climate information usability gap. Nat. Clim. Change 2, 789–794. (10.1038/nclimate1614)

[26] Dilling L, Lemos MC 2011 Creating usable science: opportunities and constraints for climate knowledge use and their implications for science policy. Glob. Environ. Change 21, 680–689. (10.1016/j.gloenvcha.2010.11.006)

[27] Hawkins RP, Kreuter M, Resnicow K, Fishbein M, Dijkstra A 2008 Understanding tailoring in communicating about health. Health Educ. Res. 23, 454–466. (10.1093/her/cyn004) 18349033 PMC3171505

[28] Schar C, Vidale PL, Luthi D, Frei C, Haberli C, Liniger MA, Appenzeller C 2004 The role of increasing temperature variability in European summer heatwaves. Nature 427, 332–336. (10.1038/nature02300) 14716318

[29] Karl TR 2009 Global climate change impacts in the United States. Cambridge, UK: Cambridge University Press.

[30] Stephens EM, Edwards TL, Demeritt D 2012 Communicating probabilistic information from climate model ensembles—lessons from numerical weather prediction. Wiley Interdiscip. Rev. Clim. Change 3, 409–426. (10.1002/wcc.187)

[31] Pidgeon N, Fischhoff B 2011 The role of social and decision sciences in communicating uncertain climate risks. Nat. Clim. Change 1, 35–41. (10.1038/nclimate1080)

[32] Stoutenborough JW, Vedlitz A 2014 The effect of perceived and assessed knowledge of climate change on public policy concerns: an empirical comparison. Environ. Sci. Policy 37, 23–33. (10.1016/j.envsci.2013.08.002)

[33] van der Linden S 2015 The social-psychological determinants of climate change risk perceptions: towards a comprehensive model. J. Environ. Psychol. 41, 112–124. (10.1016/j.jenvp.2014.11.012)

[34] Moser SC 2014 Communicating adaptation to climate change: the art and science of public engagement when climate change comes home. Wiley Interdiscip. Rev. Clim. Change 5, 337–358. (10.1002/wcc.276)

[35] Demeritt D, Langdon D 2004 The UK Climate Change Programme and communication with local authorities. Glob. Environ. Change 14, 325–336. (10.1016/j.gloenvcha.2004.06.003)

[36] Porter J, Demeritt D, Dessai S 2014 The right stuff? Informing adaptation to climate change in British local government. SRI Papers 76, 1–30.

[37] Pearce G, Cooper S 2011 Sub-national responses to climate change in England: evidence from local area agreements. Local Gov. Stud. 37, 199–217. (10.1080/03003930.2011.554825)

[38] de Oliveira JAP 2009 The implementation of climate change related policies at the subnational level: an analysis of three countries. Habitat Int. 33, 253–259. (10.1016/j.habitatint.2008.10.006)

[39] Juhola S, Westerhoff L 2011 Challenges of adaptation to climate change across multiple scales: a case study of network governance in two European countries. Environ. Sci. Policy 14, 239–247. (10.1016/j.envsci.2010.12.006)

[40] Bauer A, Feichtinger J, Steurer R 2012 The governance of climate change adaptation in 10 OECD countries: challenges and approaches. J. Environ. Pol. Plan. 14, 279–304. (10.1080/1523908x.2012.707406)

[41] Massey E, Biesbroek R, Huitema D, Jordan A 2014 Climate policy innovation: the adoption and diffusion of adaptation policies across Europe. Glob. Environ. Change 29, 434–443. (10.1016/j.gloenvcha.2014.09.002)

[42] Lorenz S, Dessai S, Paavola J, Forster P 2013 The communication of physical science uncertainty in European National Adaptation Strategies. Clim. Change 132, 143–155. (10.1007/s10584-013-0809-1) 26347116 PMC4555652

[43] Moss RH *et al* 2010 The next generation of scenarios for climate change research and assessment. Nature 463, 747–756. (10.1038/nature08823) 20148028

[44] Fagerlin A, Zikmund-Fisher BJ, Ubel PA, Jankovic A, Derry HA, Smith DM 2007 Measuring numeracy without a math test: development of the subjective numeracy scale. Med. Decis. Making 27, 672–680. (10.1177/0272989x07304449) 17641137

[45] Pallant J 2010 SPSS survival manual: a step by step guide to data analysis using SPSS, 4th edn Maidenhead, UK: McGraw-Hill/Open University Press.

[46] Daron J, Lorenz S, Wolski P, Blamey RC, Jack C In press. Interpreting climate data visualisations to inform adaptation decisions. Clim. Risk Manage. (10.1016/j.crm.2015.06.007)

[47] Gigerenzer G 1998 *Ecological intelligence: an adaptation for frequencies. The evolution of mind*, pp. 9–29. Oxford, UK: Oxford University Press.

[48] Sirota M, Juanchich M, Hagmayer Y 2014 Ecological rationality or nested sets? Individual differences in cognitive processing predict Bayesian reasoning. Psychon. Bull. Rev. 21, 198–204. (10.3758/s13423-013-0464-6) 23794254

[49] Malka A, Krosnick JA, Langer G 2009 The association of knowledge with concern about global warming: trusted information sources shape public thinking. Risk Anal. 29, 633–647. (10.1111/j.1539-6924.2009.01220.x) 19302280

[50] Milfont TL 2012 The interplay between knowledge, perceived efficacy, and concern about global warming and climate change: a one-year longitudinal study. Risk Anal. 32, 1003–1020. (10.1111/j.1539-6924.2012.01800.x) 22489642

[51] Sundblad EL, Biel A, Gärling T 2007 Cognitive and affective risk judgements related to climate change. J. Environ. Psychol. 27, 97–106. (10.1016/j.jenvp.2007.01.003)

[52] Knopman DS 2006 Success matters: recasting the relationship among geophysical, biological, and behavioral scientists to support decision making on major environmental challenges. Water Resour. Res. 42, 1–2. (10.1029/2005WR004333)

[53] Rodriguez Estrada FC, Davis LS 2015 Improving visual communication of science through the incorporation of graphic design theories and practices into science communication. Sci. Commun. 37, 140–148. (10.1177/1075547014562914)

[54] Fischhoff B 2011 Applying the science of communication to the communication of science. Clim. Change 108, 701–705. (10.1007/s10584-011-0183-9)

[55] McInerny GJ *et al* 2014 Information visualisation for science and policy: engaging users and avoiding bias. Trends Ecol. Evol. 29, 148–157. (10.1016/j.tree.2014.01.003) 24565371

[56] Lemos MC, Rood RB 2010 Climate projections and their impact on policy and practice. Wiley Interdiscip. Rev. Clim. Change 1, 670–682. (10.1002/wcc.71)

[57] Nicholls N 1999 Cognitive illusions, heuristics, and climate prediction. Bull. Am. Meteorol. Soc. 80, 1385–1397. (10.1175/1520-0477(1999)080<1385:CIHACP>2.0.CO)

[58] Rothstein H, Borraz O, Huber M 2012 Risk and the limits of governance: exploring varied patterns of risk-based governance across Europe. Regul. Gov. 7, 215–235. (10.1111/j.1748-5791.2012.01153.x)

[59] Pelling M, High C, Dearing J, Smith D 2008 Shadow spaces for social learning: a relational understanding of adaptive capacity to climate change within organisations. Environ. Plan A 40, 867–884. (10.1068/a39148)

[60] Vulturius G, Swartling ÅG 2015 Overcoming social barriers to learning and engagement with climate change adaptation: experiences with Swedish forestry stakeholders. Scand. J. For. Res. 30, 217–225. (10.1080/02827581.2014.1002218)

