# Supplementary material for: Tailoring the visual communication of climate projections for local adaptation practitioners in Germany and the UK
Source: Philos Trans A Math Phys Eng Sci. 2015 Nov 28;373(2055):20140457. doi: 10.1098/rsta.2014.0457 (PMC4608031; doi:10.1098/rsta.2014.0457)
Supplement: Questionnaire - UK [file rsta20140457supp1.pdf]

Supplementary material for [Lorenz, S., Dessai, S., Forster, P. M. and Paavola, J.], [2015], [Tailoring the visual communication of climate projections for local adaptation practitioners in Germany and the UK], *Phil. Trans. R. Soc. A.* doi: 10.1098/not yet assigned .

Questionnaire - UK

Welcome

The past year has shown us how much we can be affected by extreme weather events and how much damage such events can cause. Scientists and government produce climate projections of the future to help organisations minimise such damages under a changing future climate.

Are climate projections being communicated in a format that you can understand? Are there easier, more intuitive, ways of visualising and communicating the same information? This survey explores to what extent you understand and interpret visualisations of climate projections: are your needs being met? It is very important to understand your views and feed that back to the scientists. This research aims to improve scientific communication to help you and others better understand and interpret climate projections to enable you to create more resilient and future-proof organisations.

Who should get involved?

I am looking for people from the **business community** and the **Local Government sector**. Beyond that it doesn't matter if you are actively involved in the adaptation process within your organisation or are only just starting to think about it, are very experienced with using climate data or have always steered clear of graphs and figures, I would like to hear your opinion. It is extremely important that scientists take into account the views and perceptions of everyone who is expected to adapt to climate change when they design their communication, so all of your opinions are valued.

Project information

This survey is being conducted as part of a PhD project entitled 'Uncertainties in European climate projections and their consequences for national adaptation strategies' at the University of Leeds and is funded by the Natural Environment Research Council. This research project runs between Oct 2011 and September 2015.

Confidentiality and consent

Taking part in the survey is voluntary. If you do complete the survey you are consenting to your responses being collected and analysed. As the findings are going into a PhD thesis responses may be published, however, all data will be anonymised. I would like to reassure you that all the original data collected here will be kept in strictest confidence and will be used for research purposes only.

You have the right to withdraw at any point before submitting the survey. After that I can only withdraw your responses, up to the point when the data has been written up, if you provide a name and e-mail address at the end of the survey.

Findings and research outcomes

If you would like to know the findings from this survey and the outcomes of the further research, I would be happy to share these with you once all the data has been anonymised and written up. If you are interested please just get in touch!

© The Authors under the terms of the Creative Commons Attribution License http://creativecommons.org/licenses/by/3.0/, which permits unrestricted use, provided the original author and source are credited.

Supplementary material for [Lorenz, S., Dessai, S., Forster, P. M. and Paavola, J.], [2015], [Tailoring the visual communication of climate projections for local adaptation practitioners in Germany and the UK], *Phil. Trans. R. Soc. A.* doi: 10.1098/not yet assigned .

Contact

If you have any questions about the survey or the research project please contact, **Susanne Lorenz** *ee08sl@leeds.ac.uk*

http://www.see.leeds.ac.uk/people/s.lorenz

Keep in touch

If you find this topic interesting and would be happy to be contacted again during further stages of the research please include your contact details at the end of the survey and/or get in touch with me directly.

How long will it take?

The survey should not take more than **20 minutes** to complete.

Thank you very much for your time and your input!

© The Authors under the terms of the Creative Commons Attribution License http://creativecommons.org/licenses/by/3.0/, which permits unrestricted use, provided the original author and source are credited.

Supplementary material for [Lorenz, S., Dessai, S., Forster, P. M. and Paavola, J.], [2015], [Tailoring the visual communication of climate projections for local adaptation practitioners in Germany and the UK], *Phil. Trans. R. Soc. A.* doi: 10.1098/not yet assigned .

About you

Participant

1. Are you a participant from the

- ○ Public Sector (please answer questions in section 1 and enter 'N/A' for the questions in section 2)
- ○ Business Community (please answer questions in section 2 and enter 'N/A' for the questions section 1)

Section 1

2. Name of Public Sector employer *(Optional)*

3. Department

4. Job title

5. Name of county

Section 2

6. Name of business sector employer *(Optional)*

7. Business sector

8. Business size

- ○ Less than 10
- ○ Between 10 and 50
- ○ Between 50 - 250
- ○ More than 250

9. Job title

10. Name of county business is located in

© The Authors under the terms of the Creative Commons Attribution License http://creativecommons.org/licenses/by/3.0/, which permits unrestricted use, provided the original author and source are credited.

Supplementary material for [Lorenz, S., Dessai, S., Forster, P. M. and Paavola, J.], [2015], [Tailoring the visual communication of climate projections for local adaptation practitioners in Germany and the UK], *Phil. Trans. R. Soc. A.* doi: 10.1098/not yet assigned .

More about you

11. Your age

- Under 20
- 20 -29
- 30-39
- 40-49
- 50-59
- 60 and over

12. Your gender

- Male
- Female

13. Are you colour-blind?

- Yes
- No

14. Which qualifications do you have? Tick every box that applies if you have any of the qualifications listed.

If your UK qualification is not listed, tick the box that contains its nearest equivalent.

If you have qualifications outside the UK, tick the 'Foreign qualifications' box and the nearest UK equivalent (if known).

(select all that apply)

- [ ] 1-4 O Levels/ CSEs/ GCSEs (any grades), Entry Level, Foundation Diploma
- [ ] NVQ Level 1, Foundation GNVQ, Basic Skills
- [ ] 5+ O Levels (passes), CSEs (grade 1)/ GCSEs (Grades A*-C), School Certificate, 1 A Level/ 2-3 AS Levels/ VCEs, Higher Diploma
- [ ] NVQ Level 2, Intermediate GNVQ, City and Guilds Craft, BTEC First/ General Diploma, RSA Diploma
- [ ] Apprenticeship
- [ ] 2+ A Levels/ VCEs, 4+ AS Levels, Higher School Certificate, Progression/ Advanced Diploma
- [ ] NVQ Level 3, Advanced GNVQ, City and Guilds Advanced Craft, ONC, OND, BTEC National, RSA Advanced Diploma
- [ ] Degree (e.g. BSc, BA)

© The Authors under the terms of the Creative Commons Attribution License http://creativecommons.org/licenses/by/3.0/, which permits unrestricted use, provided the original author and source are credited.

- ☐ Higher Degree (e.g. MA, MSc, PhD, PGCE)
- ☐ NVQ Level 4-5, HNC, HND, RSA Higher Diploma, BTEC Higher Level
- ☐ Professional qualifications (e.g. teaching, accountancy)
- ☐ Other vocational, work-related qualifications
- ☐ Foreign qualifications
- ☐ No qualifications

15. How many years of work experience have you got in your profession/ job? This can include different employers, but would exclude radical career shifts (e.g. from being a chef to being a maths teacher).

- ○ 0-5 years
- ○ 6-10 years
- ○ 11-15 years
- ○ 16-20 years
- ○ 21-25 years
- ○ 26-30 years
- ○ 31-35 years
- ○ 36-40 years
- ○ 41-45 years

Visualisations of climate projections

In this section we want to see how easy to interpret and how intuitive you find different types of visualising climate change projections.

The data

The visualisations in this survey are based on monthly data from 14 global climate models created by climate modelling centres around the world, for the 2050s (2040 - 2069), under a medium emissions scenario. The data is for a 50 km x 50 km area in North East England.

© The Authors under the terms of the Creative Commons Attribution License http://creativecommons.org/licenses/by/3.0/, which permits unrestricted use, provided the original author and source are credited.

Supplementary material for [Lorenz, S., Dessai, S., Forster, P. M. and Paavola, J.], [2015], [Tailoring the visual communication of climate projections for local adaptation practitioners in Germany and the UK], *Phil. Trans. R. Soc. A.* doi: 10.1098/not yet assigned .

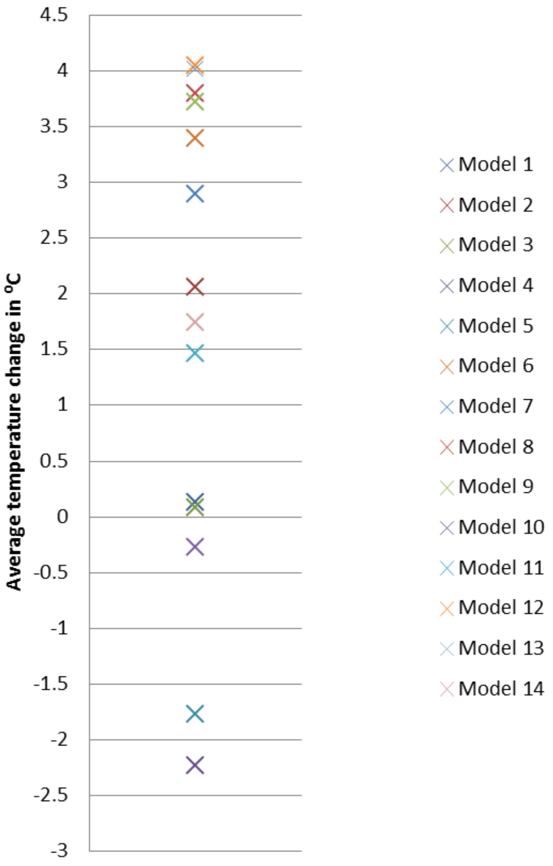

The figure illustrates the change in average summer temperatures for the 2050s (2040 - 2069) as projected by each one of the 14 global climate models under a medium emissions scenario relative to the temperatures of 1975 - 2004.

16. How many models project a decrease in summer temperature?

- 1
- 2
- 3
- 4

17. How many models project an increase in summer temperature by more than 3.0°C?

- 3
- 4
- 5
- 6

18. None of the models project a temperature change above which temperature value (to the nearest half of a degree)?

- -2.5°C

© The Authors under the terms of the Creative Commons Attribution License http://creativecommons.org/licenses/by/3.0/, which permits unrestricted use, provided the original author and source are credited.

Supplementary material for [Lorenz, S., Dessai, S., Forster, P. M. and Paavola, J.], [2015], [Tailoring the visual communication of climate projections for local adaptation practitioners in Germany and the UK], *Phil. Trans. R. Soc. A.* doi: 10.1098/not yet assigned .

- 2°C
- 4.0°C
- 4.5°C

19. Science cannot tell us which of these models is the 'correct one'. Knowing this, which temperature value do you think your organisation should plan for?

© The Authors under the terms of the Creative Commons Attribution License http://creativecommons.org/licenses/by/3.0/, which permits unrestricted use, provided the original author and source are credited.

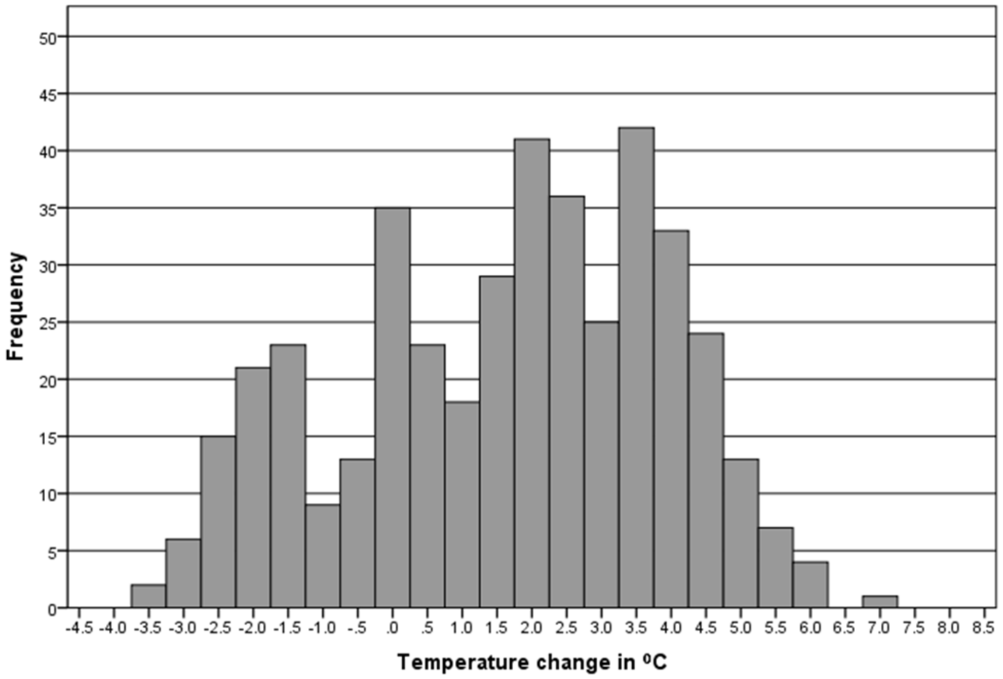

The figure illustrates the change in summer temperature for the 2050s (2040 - 2069) as projected by 14 global climate models under a medium emissions scenario relative to the temperatures of 1975 - 2004. Each bar represents a temperature range of 0.5°C (e.g. the bar marked '0°C' includes all projected temperature values between –0.25°C and 0.25°C). The focus in the figure rests on the midpoints in these ranges and the bars have been marked accordingly. The height of the bar depends on how frequently the values within its range have been projected by the models, and is thus linked to the likelihood of those values having been projected. In this plot we assume that each model is equally likely.

20. Which is the most likely temperature change projected by the models?

- -3.5°C
- 2.0°C
- 3.5°C
- 7.0°C

21. Which is the least likely temperature change projected by the models?

- -3.5°C
- 3.5°C
- 6.5°C
- 7.0°C

22. What is the range of projected temperature change in the figure?

- Between -3.5°C and 7.0°C
- Between 0°C and 4.5°C
- Between -3.5°C and 6.0°C
- Between 2.0°C and 4.0°C

© The Authors under the terms of the Creative Commons Attribution License http://creativecommons.org/licenses/by/3.0/, which permits unrestricted use, provided the original author and source are credited.

23. Which value is more likely -2.5°C or 5.0°C?

- ○ -2.5°C
- ○ 5.0°C

24. Are you more likely to get a temperature change below -2.5°C or above 5.0°C?

- ○ below -2.5°C
- ◉ above 5.0°C

© The Authors under the terms of the Creative Commons Attribution License http://creativecommons.org/licenses/by/3.0/, which permits unrestricted use, provided the original author and source are credited.

Supplementary material for [Lorenz, S., Dessai, S., Forster, P. M. and Paavola, J.], [2015], [Tailoring the visual communication of climate projections for local adaptation practitioners in Germany and the UK], *Phil. Trans. R. Soc. A.* doi: 10.1098/not yet assigned .

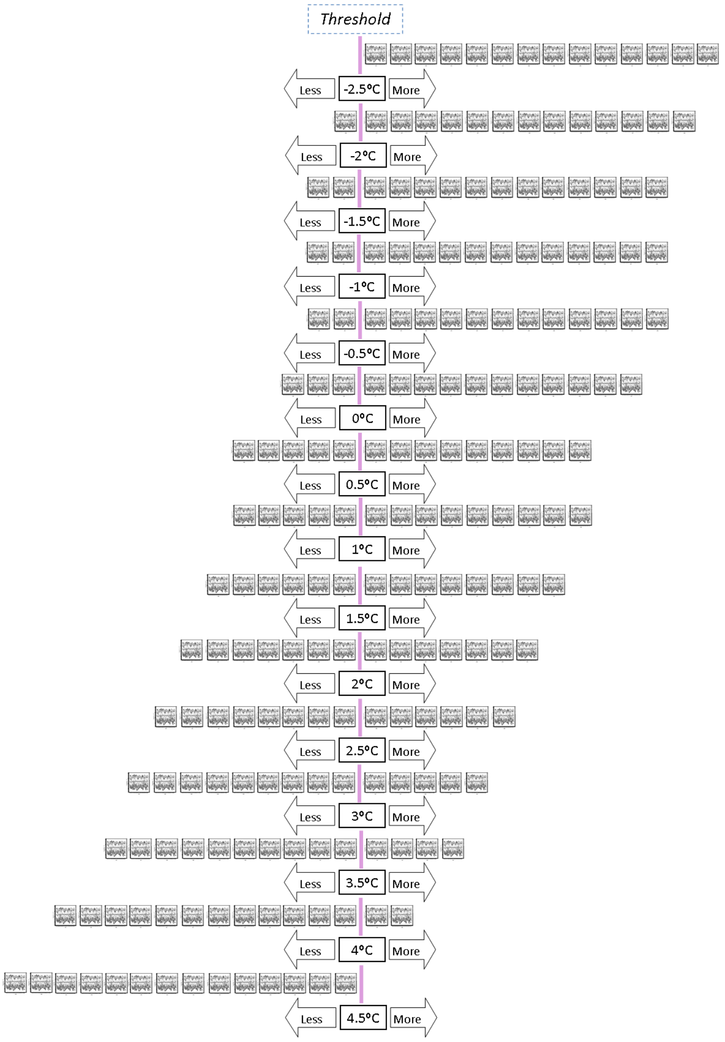

The figure illustrates the change in average summer temperatures for the 2050s (2040 - 2069) as projected by each one of the 14 global climate models under a medium emissions scenario relative to the temperatures of 1975 - 2004. The figure shows how many models project a mean change above or below a number of thresholds. Each square represents a model. The purple line in the centre of each line of squares is the threshold and the respective temperature thresholds are given in the boxes beneath the purple line.

25. How many models project a decrease in summer temperature?

- ○ 1
- ○ 2
- ○ 3
- ○ 4

26. How many models project an increase in summer temperature by more than 3.0°C?

- ○ 3
- ○ 4
- ○ 5
- ○ 6

27. None of the models project a temperature change above which temperature threshold

© The Authors under the terms of the Creative Commons Attribution License http://creativecommons.org/licenses/by/3.0/, which permits unrestricted use, provided the original author and source are credited.

(to the nearest half of a degree)?

- -2.5°C
- 2°C
- 4.0°C
- 4.5°C

28. Science cannot tell us which of these models is the 'correct one'. Knowing this, which temperature threshold do you think your organisation should plan for?

© The Authors under the terms of the Creative Commons Attribution License http://creativecommons.org/licenses/by/3.0/, which permits unrestricted use, provided the original author and source are credited.

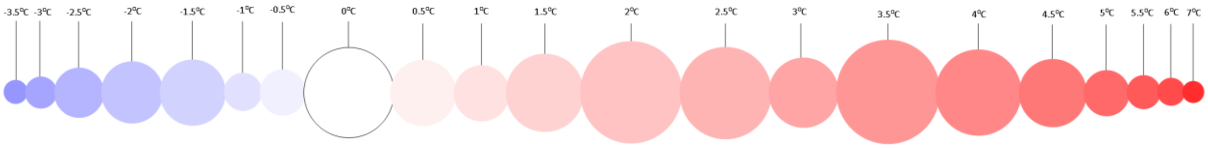

The figure illustrates the change in summer temperature for the 2050s (2040 - 2069) as projected by 14 global climate models under a medium emissions scenario relative to the temperatures of 1975 - 2004. Each bubble represents a temperature range of 0.5°C (e.g. the bubble marked '0°C' includes all projected temperature values between –0.25°C and 0.25°C). The focus in the figure rests on the midpoints in these ranges and the bubbles have been marked accordingly. The size of the bubble depends on how frequently the values within its range have been projected by the models, and is thus linked to the likelihood of those values having been projected. In this plot we assume that each model is equally likely.

29. Which is the most likely temperature change projected by the models?

- -3.5°C
- 2.0°C
- 3.5°C
- 7.0°C

30. Which is the least likely temperature change projected by the models?

- -3.5°C
- 3.5°C
- 6.5°C
- 7.0°C

31. What is the range of projected temperature change in the figure?

- Between -3.5°C and 7.0°C
- Between 0°C and 4.5°C
- Between -3.5°C and 6.0°C
- Between 2.0°C and 4.0°C

32. Which value is more likely -2.5°C or 5.0°C?

- -2.5°C
- 5.0°C

33. Are you more likely to get a temperature change below -2.5°C or above 5.0°C?

- below -2.5°C
- above 5.0°C

© The Authors under the terms of the Creative Commons Attribution License http://creativecommons.org/licenses/by/3.0/, which permits unrestricted use, provided the original author and source are credited.

Supplementary material for [Lorenz, S., Dessai, S., Forster, P. M. and Paavola, J.], [2015], [Tailoring the visual communication of climate projections for local adaptation practitioners in Germany and the UK], *Phil. Trans. R. Soc. A.* doi: 10.1098/not yet assigned .

Figure 1

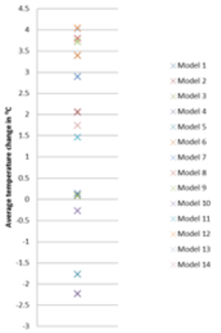

The figure illustrates the change in average summer temperatures for the 2050s (2040 - 2069) as projected by each one of the 14 global climate models under a medium emissions scenario relative to the temperatures of 1975 - 2004.

Figure 2

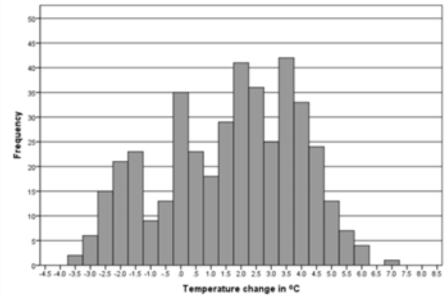

The figure illustrates the change in summer temperature for the 2050s (2040 - 2069) as projected by 14 global climate models under a medium emissions scenario relative to the temperatures of 1975 - 2004. Each bar represents a temperature range of 0.5°C (e.g. the bar marked '0°C' includes all projected temperature values between –0.25°C and 0.25°C). The focus in the figure rests on the midpoints in these ranges and the bars have been marked accordingly. The height of the bar depends on how frequently the values within its range have been projected by the models, and is thus linked to the likelihood of those values having been projected. In this plot we assume that each model is equally likely.

Figure 3

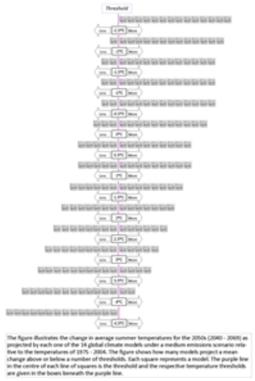

Figure 4

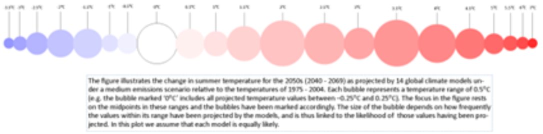

34. Which figure did you find the easiest to understand?

- Figure 1
- Figure 2
- Figure 3
- Figure 4

35. Please briefly explain your choice in the space below (e.g colour, type of graph used etc.). *(Optional)*

© The Authors under the terms of the Creative Commons Attribution License http://creativecommons.org/licenses/by/3.0/, which permits unrestricted use, provided the original author and source are credited.

Supplementary material for [Lorenz, S., Dessai, S., Forster, P. M. and Paavola, J.], [2015], [Tailoring the visual communication of climate projections for local adaptation practitioners in Germany and the UK], *Phil. Trans. R. Soc. A.* doi: 10.1098/not yet assigned .

36. Which figure do you feel is presenting the information in the most scientific way?

- Figure 1
- Figure 2
- Figure 3
- Figure 4
- All of them equally

37. Please briefly explain your choice in the space below. *(Optional)*

38. Which figure do you find the most aesthetically pleasing to the eye?

- Figure 1
- Figure 2
- Figure 3
- Figure 4

39. Please briefly explain your choice in the space below. *(Optional)*

40. If you had to make a planning decision, which of these figures would you find most helpful for your decision-making process?

- Figure 1
- Figure 2
- Figure 3
- Figure 4

© The Authors under the terms of the Creative Commons Attribution License http://creativecommons.org/licenses/by/3.0/, which permits unrestricted use, provided the original author and source are credited.

- Depends on the decision
- None of the above

41. Please briefly explain your choice in the space below. *(Optional)*

42. If you had to persuade someone in your organisation (e.g. your colleagues or your boss) of the necessity to start planning for changes in future summer temperatures, which one of these figures would you choose?

- Figure 1
- Figure 2
- Figure 3
- Figure 4
- I wouldn't use a figure at all

43. Please briefly explain your choice in the space below. *(Optional)*

© The Authors under the terms of the Creative Commons Attribution License http://creativecommons.org/licenses/by/3.0/, which permits unrestricted use, provided the original author and source are credited.

Supplementary material for [Lorenz, S., Dessai, S., Forster, P. M. and Paavola, J.], [2015], [Tailoring the visual communication of climate projections for local adaptation practitioners in Germany and the UK], *Phil. Trans. R. Soc. A.* doi: 10.1098/not yet assigned .

Knowledge, understanding and preference

The last section is about your knowledge, understanding and preferences. For each of the following questions, please check the box that best reflects your answer.

Knowledge and experience

44.

1 = ***Not at all***
6 = ***A lot***

	1	2	3	4	5	6
**a.** How much do you engage with climate projections in your day-to-day job (e.g. UK Climate Projections)?	○	○	○	○	○	○

45.

1 = ***Not at all good***
6 = ***Extremely good***

	1	2	3	4	5	6
**a.** How good is your knowledge of the topic of climate change?	○	○	○	○	○	○

46.

1 = ***Not at all***
6 = ***A lot***

	1	2	3	4	5	6
**a.** Have you been actively involved in the climate change adaptation process in your organisation?	○	○	○	○	○	○

Preference

47.

1 = ***Always prefer percentages***
6 = ***Always prefer words***

	1	2	3	4	5	6
**a.** When you hear a weather forecast, do you prefer predictions using percentages (e.g., "there will be a 20% chance of rain today") or predictions using only words (e.g., "there is a small chance of rain today")?	○	○	○	○	○	○

48.

1 = ***Always prefer words***
6 = ***Always prefer numbers***

	1	2	3	4	5	6

© The Authors under the terms of the Creative Commons Attribution License http://creativecommons.org/licenses/by/3.0/, which permits unrestricted use, provided the original author and source are credited.

**a.** When people tell you the chance of something happening, do you prefer that they use words ("it rarely happens") or numbers ("there's a 1% chance")?	○	○	○	○	○	○

49.

1 = Not at all helpful
6 = Extremely helpful

	1	2	3	4	5	6
**a.** When reading the newspaper, how helpful do you find tables and graphs that are part of a story?	○	○	○	○	○	○

50.

1 = Never
6 = Very often

	1	2	3	4	5	6
**a.** How often do you find numerical information to be useful in your line of work?	○	○	○	○	○	○

51.

1 = Never
6 = Very often

	1	2	3	4	5	6
**a.** How often do you use graphs and figures in your own work?	○	○	○	○	○	○

Understanding

52.

1 = Not at all good
6 = Extremely good

	1	2	3	4	5	6
**a.** How good are you at working with fractions?	○	○	○	○	○	○

53.

1 = Not at all good
6 = Extremely good

	1	2	3	4	5	6
**a.** How good are you at working with percentages?	○	○	○	○	○	○

54.

© The Authors under the terms of the Creative Commons Attribution License http://creativecommons.org/licenses/by/3.0/, which permits unrestricted use, provided the original author and source are credited.

	**1**	**2**	**3**	**4**	**5**	**6**
			**1 =** ***Not at all good*** **6 =** ***Extremely good***			
**a.** How good are you at figuring out how much a shirt will cost if it is 25% off?	○	○	○	○	○	○

55.

	**1**	**2**	**3**	**4**	**5**	**6**
			**1 =** ***Not at all good*** **6 =** ***Extremely good***			
**a.** How good are you at calculating a 15% tip?	○	○	○	○	○	○

Your comments and feedback

56. If you have any additional thoughts or suggestions, whether that is on the survey, other examples of visualising information that you really like or comments on the research, I would love to hear them. Please use the space below or get in touch with me directly (ee08sl@leeds.ac.uk) *(Optional)*

© The Authors under the terms of the Creative Commons Attribution License http://creativecommons.org/licenses/by/3.0/, which permits unrestricted use, provided the original author and source are credited.

Supplementary material for [Lorenz, S., Dessai, S., Forster, P. M. and Paavola, J.], [2015], [Tailoring the visual communication of climate projections for local adaptation practitioners in Germany and the UK], *Phil. Trans. R. Soc. A.* doi: 10.1098/not yet assigned .

The University of Leeds will be conducting further research in this area during the next 24 months, if you would be happy to be contacted in the near future as part of a follow-up to this questionnaire to discuss some of the issues raised about climate change communication, please provide contact details below.

Your details

57. Name *(Optional)*

58. Email address *(Optional)*

59. Phone *(Optional)*

End of the survey

Thank you for your time and for taking part in this survey. Your participation is much appreciated!

If you would like to discuss this topic with me further please do not hesitate to get in touch:

Susanne Lorenz *ee08sl@leeds.ac.uk*

If you would like to find out more about my research please go to:

http://www.see.leeds.ac.uk/people/s.lorenz

© The Authors under the terms of the Creative Commons Attribution License http://creativecommons.org/licenses/by/3.0/, which permits unrestricted use, provided the original author and source are credited.
