# Supplementary material for: Tailoring the visual communication of climate projections for local adaptation practitioners in Germany and the UK
Source: Philos Trans A Math Phys Eng Sci. 2015 Nov 28;373(2055):20140457. doi: 10.1098/rsta.2014.0457 (PMC4608031; doi:10.1098/rsta.2014.0457)
Supplement: Questionnaire - Germany [file rsta20140457supp2.pdf]

Supplementary material for [Lorenz, S., Dessai, S., Forster, P. M. and Paavola, J.], [2015], [Tailoring the visual communication of climate projections for local adaptation practitioners in Germany and the UK], *Phil. Trans. R. Soc. A.* doi: 10.1098/not yet assigned .

Questionnaire - Germany

Herzlich Willkommen!

Das letzte Jahr hat uns gezeigt, wie sehr uns Extremwetterereignisse beeinflussen können und wie viel Schaden solche Ereignisse verursachen können. Wissenschaftler und die Regierung erstellen Klimaprojektionen für die Zukunft, um Organisationen dabei zu helfen, solche Schäden unter einem zukünftigem Klimawandel zu vermeiden.

Werden diese Klimaprojektionen in einer Art und Weise kommuniziert, die Sie verstehen? Gibt es einfachere, intuitivere Wege dieselben Informationen zu visualisieren und zu kommunizieren? Dieses Forschungsprojekt zielt darauf ab, wissenschaftliche Kommunikation zu verbessern and Ihnen die Informationen aus der Wissenschaft zum Thema Klimawandel in einer verständlichen Art und Weise näher zu bringen, sodass Sie die Risiken der möglichen Folgen für Ihre Organisationen abschätzen können.

Wer sollte sich beteiligen?

Dieses Forschungsprojekt richtet sich insbesondere an Personen, die im Amts- oder Verwaltungswesen arbeiten, sei es bei der Stadt, der Gemeinde, dem Landkreis, oder beim Land. Ob Sie aktiv bei Ihrer Organisation im Klimaanpassungsprozess involviert sind oder gerade erst anfangen sich mit dem Thema zu beschäftigen, ist nicht von Bedeutung. Kern der Sache ist es, Ihre Meinung zu hören, denn Wissenschaftler müssen diese kennen, wenn sie mit Ihnen kommunizieren wollen.

Information zum Forschungsprojekt

Diese Befragung wird als Teil einer Doktorarbeit mit dem Titel "Unsicherheiten in europäischen Klimaprojektionen und deren Auswirkungen auf nationale Anpassungsstrategien" an der University of Leeds, Großbritannien, durchgeführt und wird durch den Forschungsrat für die natürliche Umwelt (Natural Environment Research Council) in Großbritannien finanziert. Das Forschungsprojekt läuft von Oktober 2011 bis September 2015.

Einverständnis und Vertraulichkeit

Die Teilnahme an dieser Befragung ist freiwillig. Wenn Sie diese Befragung ausfüllen, stimmen Sie der Erfassung und Analyse Ihrer Antworten zu. Da die Ergebnisse in eine Doktorarbeit eingebunden werden, werden Antworten eventuell veröffentlicht. Natürlich werden alle Daten vorher anonymisiert. Ich versichere Ihnen, dass alle hier gesammelten Originaldaten vertraulich behandelt und nur zu Forschungszwecken verwendet werden.
Es steht Ihnen frei, die Befragung jederzeit im Laufe der Bearbeitung abzubrechen, wenn Sie es wünschen. In diesem Fall, kann das Forschungsprojekt nur dann auf die bereits eingegebenen Daten zugreifen, wenn Sie einen Namen und eine Emailadresse am Ende der Befragung hinterlassen.

Supplementary material for [Lorenz, S., Dessai, S., Forster, P. M. and Paavola, J.], [2015], [Tailoring the visual communication of climate projections for local adaptation practitioners in Germany and the UK], *Phil. Trans. R. Soc. A.* doi: 10.1098/not yet assigned .

Forschungsergebnisse

Falls Sie Interesse an den Resultaten der Befragung bzw. dem gesamten Forschungsprojekt haben, teile ich Ihnen diese sehr gern mit, sobald alle Daten anonymisiert und ausgewertet sind. In diesem Fall, können Sie mich, Susanne Lorenz, gerne der unten genannten Emailadresse kontaktieren.

Kontakt

Wenn Sie Fragen zur Erhebung oder zum Forschungsprojekt haben, kontaktieren Sie bitte **Susanne Lorenz** unter *ee08sl@leeds.ac.uk*.

http://www.see.leeds.ac.uk/people/s.lorenz

Bleiben Sie in Verbindung

Falls Sie dieses Thema interessant finden und es Ihnen nichts ausmachen würde, nochmals für die nächsten Forschungsphasen kontaktiert zu werden, dann hinterlassen Sie bitte Ihre Kontaktdaten am Ende der Befragung oder setzen Sie sich direkt mit mir in Verbindung.

Navigation

Um zur nächsten Frage zu gelangen, drücken Sie bitte die **Weiter**-Taste. Bitte verwenden Sie ab diesem Zeitpunkt die Vor- und Zurücktasten in Ihrem Browserfenster nicht mehr, denn das wird die Befragung abbrechen. Erst wenn die Befragung beendet ist und die letzte Seite erreicht wurde, werden alle Antworten automatisch gespeichert. Falls Sie vorher abbrechen, wird keine Ihrer Antworten gespeichert sein.

Herunterladen der grafischen Darstellungen

Es kann vorkommen, dass Ihr Internetbrowser Abbildungen als „unsichere Elemente" erkennt und Sie fragt, ob Sie diese herunterladen wollen. Nur wenn Sie dies akzeptieren, werden Sie die grafischen Darstellungen auch sehen können. Hervorgerufen wird diese Meldung durch bestimmte IT-Einstellungen in Ihrer Organisation.

Wie lange dauert die Befragung?

Die Befragung wird Sie nicht mehr als 25 Minuten Ihrer wertvollen Zeit berauben.

Vielen Dank, dass Sie sich die Zeit nehmen, dieses Forschungsprojekt zu unterstützen.

Supplementary material for [Lorenz, S., Dessai, S., Forster, P. M. and Paavola, J.], [2015], [Tailoring the visual communication of climate projections for local adaptation practitioners in Germany and the UK], *Phil. Trans. R. Soc. A.* doi: 10.1098/not yet assigned .

Über Sie

1. Arbeiten Sie

- Im öffentlichen Dienst (Gemeindeebene)
- Im öffentlichen Dienst (Landesebene)
- Im öffentlichen Dienst (Bundesebene)
- In der Forschung
- Für ein Privatunternehmen
- Sonstige(s) *(Bitte machen Sie nähere Angaben)*:

2. Für welche(s) Organisation/ Amt/ Einrichtung arbeiten Sie? *(Beantwortung freigestellt)*

3. In welcher Abteilung arbeiten Sie? *(Beantwortung freigestellt)*

4. Als was sind Sie tätig?

5. In welchem Bundesland arbeiten Sie?

6. Wie viele Angestellte hat die Organisation/ das Amt/ die Einrichtung für welche(s) Sie arbeiten? *(Beantwortung freigestellt)*

- Weniger als 10
- Zwischen 10 und 50
- Zwischen 50 und 250
- Mehr als 250

7. Wie alt sind Sie?

- Unter 20
- 20 -29
- 30-39
- 40-49
- 50-59

- 60 und älter

8. Welches Geschlecht haben Sie?

- Männlich
- Weiblich

9. Sind Sie farbenblind?

- Ja
- Nein
- Ich bin mir nicht sicher

10. Welche Abschlüsse haben Sie erzielt? Kreuzen Sie bitte alle Antworten an, die auf Sie zutreffen.

Ordnen Sie bitte im Ausland erworbene Abschlüsse einem gleichwertigen deutschen Abschluss zu.

(Wählen Sie alle zutreffenden)

- Abschluss nach höchstens 7 Jahren Schulbesuch (insbesondere Abschluss im Ausland)
- Haupt-/Volksschulabschluss
- Realschulabschluss (Mittlere Reife), Abschluss an der Polytechnischen Oberschule oder gleichwertiger Abschluss
- Fachhochschulreife
- Allgemeine oder fachgebundene Hochschulreife
- Anlernausbildung oder berufliches Praktikum von mindestens 12 Monaten
- Berufsvorbereitungsjahr
- Lehre, Berufsausbildung im dualen System
- Vorbereitungsdienst für den mittleren Dienst in der öffentlichen Verwaltung
- Berufsqualifizierender Abschluss an einer Berufsfachschule/Kollegschule, Abschluss einer 1-jährigen Schule des Gesundheitswesens
- 2- oder 3-jährige Schule des Gesundheitswesens (z. B. Krankenpflege, PTA, MTA)
- Fachschulabschluss (Meister/-in, Techniker/-in oder gleichwertiger Abschluss)
- Berufsakademie, Fachakademie
- Abschluss an einer Verwaltungsfachhochschule
- Fachhochschulabschluss, auch Ingenieurschulabschluss
- Abschluss einer Universität/ wissenschaftlichen Hochschule/ Kunsthochschule

- ☐ Promotion
- ☐ Kein Abschluss

11. Wie viele Jahre an Arbeitserfahrung haben Sie in Ihrem Beruf? Das schließt unterschiedliche Arbeitgeber mit ein. Drastische Berufswechel sind davon ausgeschlossen (z.B. ein Berufswechsel vom Koch zum Mathelehrer).

- ○ 0-5 Jahre
- ○ 6-10 Jahre
- ○ 11-15 Jahre
- ○ 16-20 Jahre
- ○ 21-25 Jahre
- ○ 31-35 Jahre
- ○ 36-40 Jahre
- ○ 41-45 Jahre

Grafische Darstellung von Klimaprojektionen

In diesem Teil der Befragung möchten wir sehen, wie einfach es für Sie ist verschiedene grafische Darstellungen von Klimaprojektionen zu interpretieren und wie erfassbar Sie diese finden.

Die Klimadaten

Die grafischen Darstellungen in dieser Erhebung basieren auf monatlichen Projektionen von 14 globalen Klimamodellen für die Mitte dieses Jahrhunderts (2040 - 2069) unter der Annahme einer gemäßigten zukünftigen Entwicklung von Treibhauskonzentrationen in der Atmosphere. Diese wurden von Klimamodellierzentren rund um den Globus erstellt. Die Daten beziehen sich auf ein 50 km mal 50 km großes Gebiet im Nordosten von Deutschland.

Supplementary material for [Lorenz, S., Dessai, S., Forster, P. M. and Paavola, J.], [2015], [Tailoring the visual communication of climate projections for local adaptation practitioners in Germany and the UK], *Phil. Trans. R. Soc. A.* doi: 10.1098/not yet assigned .

1. Abbildung

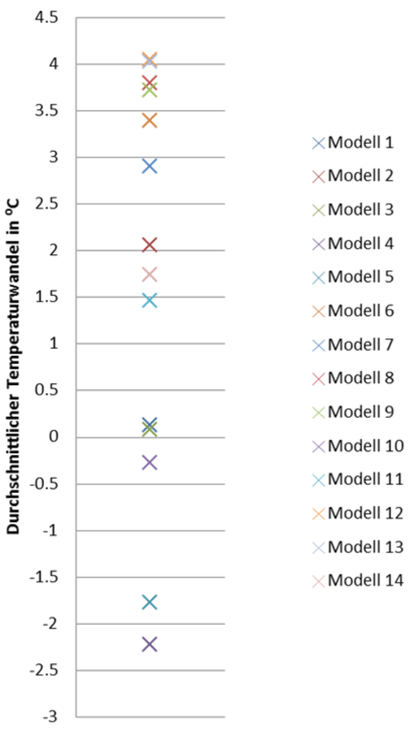

Die Abbildung zeigt den Wandel der Sommermitteltemperatur für die Mitte dieses Jahrhunderts (2040 – 2069) im Vergleich zu den Temperaturen von 1975 – 2004, wie er von jedem der 14 globalen Klimamodelle unter der Annahme einer gemäßigten zukünftigen Entwicklung von Treibhausgaskonzentrationen in der Atmosphere simuliert wird.

12. Wie viele Modelle zeigen eine Abnahme der Sommermitteltemperaturen?

- 1
- 2
- 3
- 4

13. Wie viele Modelle zeigen einen Anstieg der Sommermitteltemperaturen von mehr als 3.0°C?

- 3
- 4
- 5
- 6

14. Keines der Klimamodelle zeigt einen Temperaturwandel über welcher Temperatur (zum nächstgelegenen halben Grad gerundet)?

- -2.5°C
- 2°C
- 4.0°C
- 4.5°C

15. Die Wissenschaft kann uns nicht sagen, welches dieser Modelle richtig liegt. Mit diesem Wissen im Hinterkopf, für welchen Temperaturwert sollte Ihre Organisation, Ihrer Meinung nach, planen?

16. Was erwarten Sie, wie sich die Sommermitteltemperatur entsprechend dieser Abbildung in der Zukunft ändern wird?

- Hohe Wahrscheinlichkeit einer Abnahme
- Mittlere Wahrscheinlichkeit einer Abnahme
- Niedrige Wahrscheinlichkeit einer Abnahme
- Keine Veränderung in der Zukunft
- Hohe Wahrscheinlichkeit eines Anstiegs
- Mittlere Wahrscheinlichkeit eines Anstiegs
- Niedrige Wahrscheinlichkeit eines Anstiegs

17.

1 = *Gar nicht überzeugt*
6 = *Äußerst überzeugt*

	**1**	**2**	**3**	**4**	**5**	**6**
**a.** Wie überzeugt sind Sie von Ihrer Antwort zur vorherigen Frage?	○	○	○	○	○	○

Supplementary material for [Lorenz, S., Dessai, S., Forster, P. M. and Paavola, J.], [2015], [Tailoring the visual communication of climate projections for local adaptation practitioners in Germany and the UK], *Phil. Trans. R. Soc. A.* doi: 10.1098/not yet assigned .

2. Abbildung

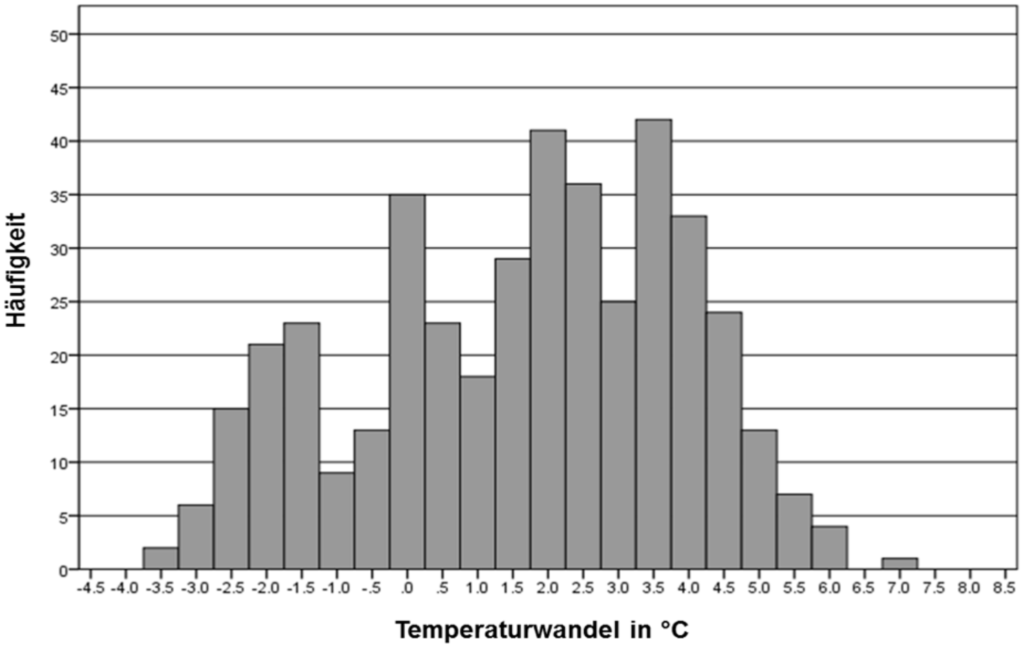

Die Abbildung zeigt den Wandel der Sommermitteltemperatur für die Mitte dieses Jahrhunderts (2040 – 2069) im Vergleich zu den Temperaturen von 1975 – 2004, wie er von jedem der 14 globalen Klimamodelle unter der Annahme einer gemäßigten zukünftigen Entwicklung von Treibhausgaskonzentrationen in der Atmosphere simuliert wird. Jeder Balken stellt eine Temperaturspanne von 0.5°C dar (z.B. der Balken der als '0°C' gekennzeichnet ist, beinhaltet alle simulierten Temperaturwerte zwischen –0.25°C und 0.25°C). Der Fokus in dieser Abbildung ist auf den Mittelpunkten in diesen Spannen und die Balken wurden dementsprechend gekennzeichnet. Die Höhe der Balken hängt davon ab, wie häufig die Werte in ihrer Spanne von den Modellen simuliert wurden, und ist somit mit der Wahrscheinlichkeit der Projektion dieser Werte verbunden. In dieser Abbildung nehmen wir an, dass jedes Modell die gleiche Wahrscheinlichkeit hat.

18. Basierend auf den Projektionen der Modelle, welchen Temperaturwandel halten Sie für den Wahrscheinlichsten?

- -3.5°C
- 2.0°C
- 3.5°C
- 7.0°C

19. Basierend auf den Projektionen der Modelle, welchen Temperaturwandel halten Sie für den Unwahrscheinlichsten?

- -3.5°C
- 3.5°C
- 6.5°C
- 7.0°C

20. Welche Wertespanne zeigen die Klimamodelle in der Abbildung?

- Zwischen -3.5°C und 7.0°C
- Zwischen 0°C und 4.5°C
- Zwischen -3.5°C und 6.0°C
- Zwischen 2.0°C und 4.0°C

21. Welcher Wert ist wahrscheinlicher: -2.5°C oder 5.0°C?

- -2.5°C
- 5.0°C

22. Ist ein Temperaturwandel unter -2.5°C oder über 5.0°C wahrscheinlicher?

- unter -2.5°C
- über 5.0°C

23. Was erwarten Sie, wie sich die Sommermitteltemperatur entsprechend dieser Abbildung in der Zukunft ändern wird?

- Hohe Wahrscheinlichkeit einer Abnahme
- Mittlere Wahrscheinlichkeit einer Abnahme
- Niedrige Wahrscheinlichkeit einer Abnahme
- Keine Veränderung in der Zukunft
- Hohe Wahrscheinlichkeit eines Anstiegs
- Mittlere Wahrscheinlichkeit eines Anstiegs
- Niedrige Wahrscheinlichkeit eines Anstiegs

24.

1 = ***Gar nicht überzeugt***
6 = ***Äußerst überzeugt***

	1	2	3	4	5	6
**a.** Wie überzeugt sind Sie von Ihrer Antwort zur vorherigen Frage?	○	○	○	○	○	○

Supplementary material for [Lorenz, S., Dessai, S., Forster, P. M. and Paavola, J.], [2015], [Tailoring the visual communication of climate projections for local adaptation practitioners in Germany and the UK], *Phil. Trans. R. Soc. A.* doi: 10.1098/not yet assigned .

3. Abbildung

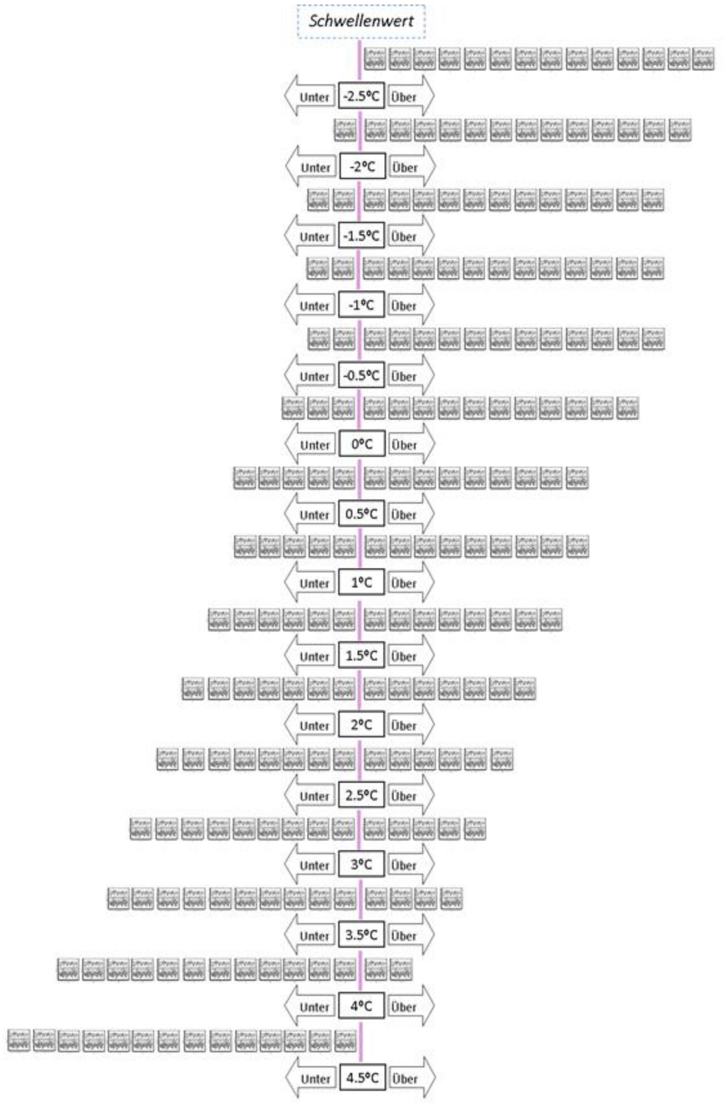

Die Abbildung zeigt den Wandel der Sommermitteltemperatur für die Mitte dieses Jahrhunderts (2040 – 2069) im Vergleich zu den Temperaturen von 1975 – 2004, wie er von jedem der 14 globalen Klimamodelle unter der Annahme einer gemäßigten zukünftigen Entwicklung von Treibhausgaskonzentrationen in der Atmosphere simuliert wird. Die Abbildung zeigt wie viele Modelle einen durchschnittlichen Wandel unter oder über einer Anzahl von Schwellenwerten simulieren. Jedes Quadrat repräsentiert ein Modell. Die lila Linie in der Mitte jeder Quadratreihe ist der Schwellenwert und der jeweilige Temperaturwert ist in dem Kasten unter der lila Linie angegeben.

25. Wie viele Modelle zeigen eine Abnahme der Sommermitteltemperaturen?

- ○ 1
- ○ 2
- ○ 3
- ○ 4

26. Wie viele Modelle zeigen einen Anstieg der Sommermitteltemperaturen von mehr als 3.0°C?

- 3
- 4
- 5
- 6

27. Keines der Klimamodelle zeigt einen Temperaturwandel über welcher Temperatur (zum nächstgelegenen halben Grad gerundet)?

- -2.5°C
- 2°C
- 4.0°C
- 4.5°C

28. Die Wissenschaft kann uns nicht sagen, welches dieser Modelle richtig liegt. Mit diesem Wissen im Hinterkopf, für welchen Temperaturwert sollte Ihre Organisation, Ihrer Meinung nach planen?

29. Was erwarten Sie, wie sich die Sommermitteltemperatur entsprechend dieser Abbildung in der Zukunft ändern wird?

- Hohe Wahrscheinlichkeit einer Abnahme
- Mittlere Wahrscheinlichkeit einer Abnahme
- Niedrige Wahrscheinlichkeit einer Abnahme
- Keine Veränderung in der Zukunft
- Hohe Wahrscheinlichkeit eines Anstiegs
- Mittlere Wahrscheinlichkeit eines Anstiegs
- Niedrige Wahrscheinlichkeit eines Anstiegs

30.

1 = *Gar nicht überzeugt*
6 = *Äußerst überzeugt*

	**1**	**2**	**3**	**4**	**5**	**6**
**a.** Wie überzeugt sind Sie von Ihrer Antwort zur vorherigen Frage?	○	○	○	○	○	○

Supplementary material for [Lorenz, S., Dessai, S., Forster, P. M. and Paavola, J.], [2015], [Tailoring the visual communication of climate projections for local adaptation practitioners in Germany and the UK], *Phil. Trans. R. Soc. A.* doi: 10.1098/not yet assigned .

4. Abbildung

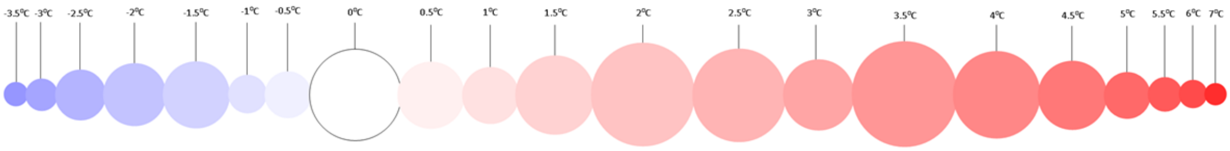

Die Abbildung zeigt den Wandel der Sommermitteltemperatur für die Mitte dieses Jahrhunderts (2040 – 2069) im Vergleich zu den Temperaturen von 1975 – 2004, wie er von jedem der 14 globalen Klimamodelle unter der Annahme einer gemäßigten zukünftigen Entwicklung von Treibhausgaskonzentrationen in der Atmosphere simuliert wird. Jede Blase stellt eine Temperaturspanne von 0.5°C dar (z.B. die Blase die als '0°C' gekennzeichnet ist, beinhaltet alle simulierten Temperaturwerte zwischen –0.25°C und 0.25°C). Der Fokus in dieser Abbildung ist auf den Mittelpunkten in diesen Spannen und die Blasen wurden dementsprechend gekennzeichnet. Die Größe der Blase hängt davon ab, wie häufig die Werte in ihrer Spanne von den Modellen simuliert wurden, und ist somit mit der Wahrscheinlichkeit der Projektion dieser Werte verbunden. In dieser Abbildung nehmen wir an, dass jedes Modell die gleiche Wahrscheinlichkeit hat.

31. Basierend auf den Projektionen der Modelle, welchen Temperaturwandel halten Sie für den Wahrscheinlichsten?

- -3.5°C
- 2.0°C
- 3.5°C
- 7.0°C

32. Basierend auf den Projektionen der Modelle, welchen Temperaturwandel halten Sie für den Unwahrscheinlichsten?

- -3.5°C
- 3.5°C
- 6.5°C
- 7.0°C

33. Welche Wertespanne zeigen die Klimamodelle in der Abbildung?

- Zwischen -3.5°C und 7.0°C
- Zwischen 0°C und 4.5°C
- Zwischen -3.5°C und 6.0°C
- Zwischen 2.0°C und 4.0°C

34. Welcher Wert ist wahrscheinlicher: -2.5°C oder 5.0°C?

- -2.5°C
- 5.0°C

35. Ist ein Temperaturwandel unter -2.5°C oder über 5.0°C wahrscheinlicher?

- unter -2.5°C
- über 5.0°C

36. Was erwarten Sie, wie sich die Sommermitteltemperatur entsprechend dieser Abbildung in der Zukunft ändern wird?

- Hohe Wahrscheinlichkeit einer Abnahme
- Mittlere Wahrscheinlichkeit einer Abnahme
- Niedrige Wahrscheinlichkeit einer Abnahme
- Keine Veränderung in der Zukunft
- Hohe Wahrscheinlichkeit eines Anstiegs
- Mittlere Wahrscheinlichkeit eines Anstiegs
- Niedrige Wahrscheinlichkeit eines Anstiegs

37.

1 = *Gar nicht überzeugt*
6 = *Äußerst überzeugt*

	**1**	**2**	**3**	**4**	**5**	**6**
**a.** Wie überzeugt sind Sie von Ihrer Antwort zur vorherigen Frage?	○	○	○	○	○	○

Supplementary material for [Lorenz, S., Dessai, S., Forster, P. M. and Paavola, J.], [2015], [Tailoring the visual communication of climate projections for local adaptation practitioners in Germany and the UK], *Phil. Trans. R. Soc. A.* doi: 10.1098/not yet assigned .

Vergleich der Abbildungen

Abbildung 1

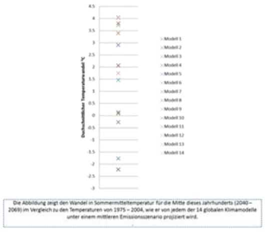

Abbildung 2

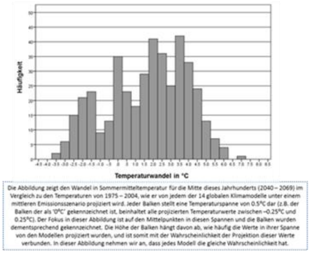

Abbildung 3

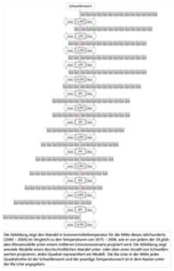

Abbildung 4

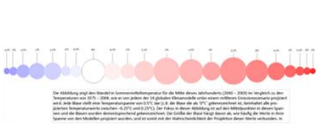

38. Welche der Abbildungen fanden Sie am einfachsten zu verstehen?

- ○ Abbildung 1
- ○ Abbildung 2
- ○ Abbildung 3
- ○ Abbildung 4

39. Bitte erläutern Sie hier kurz Ihre Wahl (z.B. Farbe, Art der benutzten Abbildung etc.) *(Beantwortung freigestellt)*

40. Welche Abbildung repräsentiert, Ihrer Meinung nach, die Information in der wissenschaflichsten Art und Weise?

- Abbildung 1
- Abbildung 2
- Abbildung 3
- Abbildung 4
- Alle vier gleichwertig

41. Bitte erläutern Sie hier kurz Ihre Wahl. *(Beantwortung freigestellt)*

42. Welche Abbildung finden Sie am ästhetischsten?

- Abbildung 1
- Abbildung 2
- Abbildung 3
- Abbildung 4

43. Bitte erläutern Sie hier kurz Ihre Wahl. *(Beantwortung freigestellt)*

44. Welche dieser Abbildung würden Sie für Ihren Entscheidungsprozess am hilfreichsten finden, wenn Sie eine Planungsentscheidung treffen müssten?

- Abbildung 1
- Abbildung 2
- Abbildung 3
- Abbildung 4
- Das hängt von der Entscheidung ab
- Keine der hier gezeigten Abbildungen

45. Bitte erläutern Sie hier kurz Ihre Wahl. *(Beantwortung freigestellt)*

46. Welche dieser Abbildungen würden Sie auswählen, wenn Sie jemanden (z.B. einen Kollegen oder Vorgesetzten) in Ihrer Organisation davon überzeugen müssten, dass es nötig ist, anzufangen für einen Wandel in zukünftigen Sommertemperaturen zu planen?

- Abbildung 1
- Abbildung 2
- Abbildung 3
- Abbildung 4
- Ich würde überhaupt keine Abbildung benutzen

47. Bitte erläutern Sie hier kurz Ihre Wahl. *(Beantwortung freigestellt)*

48. Falls diese grafischen Darstellungen zur Kommunikation von Klimaprojektionen benutzt werden würden, wie sehr würden Sie der Zuverlässigkeit der zugrunde liegenden Informationen vertrauen?

1 = *Gar nicht*
6 = *Äußerst viel*

	1	2	3	4	5	6
**a.** Abbildung 1	◯	◯	◯	◯	◯	◯
**b.** Abbildung 2	◯	◯	◯	◯	◯	◯
**c.** Abbildung 3	◯	◯	◯	◯	◯	◯
**d.** Abbildung 4	◯	◯	◯	◯	◯	◯

Wissen, Verständnis und Präferenz

Der letzte Teil dreht sich um Ihr Wissen, Ihr Verständnis und Ihre Präferenzen. Kreuzen Sie bitte für jede der folgenden Fragen das an, was am besten auf Sie zutrifft.

Wissen und Erfahrung

49.

1 = *Nie*
6 = *Sehr viel*

	1	2	3	4	5	6
**a.** Wie viel beschäftigen Sie sich mit Klimaprojektionen in Ihrem täglichen Beruf?	◯	◯	◯	◯	◯	◯

50.

1 = *Gar nicht gut*
6 = *Äußerst gut*

	1	2	3	4	5	6
**a.** Wie gut ist Ihr Wissen zum Thema Klimawandel?	◯	◯	◯	◯	◯	◯

51.

1 = *Gar nicht*
6 = *Sehr viel*

	1	2	3	4	5	6
**a.** Sind Sie aktiv im Klimaanpassungsprozess in Ihrer	◯	◯	◯	◯	◯	◯

Organisation eingebunden?

Präferenzen

52.

	**1 = *Bevorzuge immer Prozente*<br>6 = *Bevorzuge immer Wörter***					
	**1**	**2**	**3**	**4**	**5**	**6**
**a.** Wenn Sie den Wetterbericht hören, bevorzugen Sie dann Vorhersagen mit Prozenten (z.B. ‚es gibt eine 20% Chance, dass es heute regnet') oder Vorhersagen die nur Wörter benutzen (z.B. ‚es gibt eine geringe Chance, dass es heute regnet')?	○	○	○	○	○	○

53.

	**1 = *Bevorzuge immer Wörter*<br>6 = *Bevorzuge immer Nummern***					
	**1**	**2**	**3**	**4**	**5**	**6**
**a.** Wenn Ihnen die Chance, dass etwas passiert, gesagt wird, bevorzugen Sie dann Wörter (‚es passiert selten') oder Nummern (‚es gibt eine 1% Chance')?	○	○	○	○	○	○

54.

	**1 = *Gar nicht hilfreich*<br>6 = *Äußerst hilfreich***					
	**1**	**2**	**3**	**4**	**5**	**6**
**a.** Wenn Sie die Zeitung lesen, wie hilfreich finden Sie Tabellen und Graphen, die Teil einer Geschichte sind?	○	○	○	○	○	○

55.

	**1 = *Nie*<br>6 = *Sehr oft***					
	**1**	**2**	**3**	**4**	**5**	**6**
**a.** Wie oft finden Sie numerische Informationen in Ihrer Arbeit	○	○	○	○	○	○

nützlich?

56.

	**1 = *Nie*<br>6 = *Sehr oft***					
	**1**	**2**	**3**	**4**	**5**	**6**
**a.** Wie oft benutzen Sie Graphen und Abbildungen in Ihrer eigenen Arbeit?	○	○	○	○	○	○

57. Es gibt unterschiedliche Wege die Unsicherheiten in Klimaprojektionen zu kommunizieren.

	**1 = *Gar nicht nützlich*<br>6 = *Äußerst nützlich***					
	**1**	**2**	**3**	**4**	**5**	**6**
**a.** Wie nützlich finden Sie verbale Beschreibungen (z.B. Wahrscheinlichkeitsaussagen)?	○	○	○	○	○	○
**b.** Wie nützlich finden Sie numerische Beschreibungen (z.B. Prozente, Wertebereiche)?	○	○	○	○	○	○
**c.** Wie nützlich finden Sie grafische Darstellungen (z.B. Abbildungen und Graphen)?	○	○	○	○	○	○

Verständnis

58.

	**1 = *Gar nicht gut*<br>6 = *Äußerst gut***					
	**1**	**2**	**3**	**4**	**5**	**6**
**a.** Als wie gut würden Sie sich beim Arbeiten mit Brüchen einschätzen?	○	○	○	○	○	○

59.

	**1 = *Gar nicht gut*<br>6 = *Äußerst gut***					
	**1**	**2**	**3**	**4**	**5**	**6**

**a.** Als wie gut würden Sie sich beim Arbeiten mit Prozenten einschätzen?	○	○	○	○	○	○

60.

1 = ***Gar nicht gut***
6 = ***Äußerst gut***

	**1**	**2**	**3**	**4**	**5**	**6**
**a.** Als wie gut würden Sie sich dabei einschätzen, auszurechnen wie viel ein Hemd mit 25% Rabatt kostet?	○	○	○	○	○	○

61.

1 = ***Gar nicht gut***
6 = ***Äußerst gut***

	**1**	**2**	**3**	**4**	**5**	**6**
**a.** Als wie gut würden Sie sich dabei einschätzen, ein Trinkgeld von 15% auszurechnen?	○	○	○	○	○	○

Ihre Kommentare und Feedback

62. Falls Sie noch weitere Gedanken oder Vorschläge haben, sei es zur Befragung, Beispiele zur grafischen Darstellungen von Informationen, die Ihnen besonders gefallen oder Kommentare zu dieser Forschung, teilen Sie mir diese gerne mit. Bitte nutzen Sie dieses Feld oder kontaktieren Sie mich direkt (ee08sl@leeds.ac.uk) *(Beantwortung freigestellt)*

Supplementary material for [Lorenz, S., Dessai, S., Forster, P. M. and Paavola, J.], [2015], [Tailoring the visual communication of climate projections for local adaptation practitioners in Germany and the UK], *Phil. Trans. R. Soc. A.* doi: 10.1098/not yet assigned .

Die University of Leeds wird in den kommenden 24 Monate noch weitere Forschungsaktivitäten in diesem Bereich betreiben. Ich würde mich freuen, wenn ich Sie auch zukünftig zur Weiterverfolgung dieser Befragung, kontaktieren darf, um manche dieser Themenpunkte im Bereich Klimakommunikation zu diskutieren. Sollten Sie einverstanden sein, dann bitte ich Sie hier Ihre Kontaktdaten zu hinterlassen. Ihre Antworten in der Befragung bleiben natürlich trotzdem anonym and die Angabe der Kontaktdaten ist davon unabhängig.

Ihre Angaben

63. Name *(Beantwortung freigestellt)*

64. Emailadresse *(Beantwortung freigestellt)*

65. Telefonnummer *(Beantwortung freigestellt)*

Ende der Befragung

Vielen Dank, dass Sie sich die Zeit genommen haben, an dieser Befragung teilzunehmen.

Falls Sie dieses Thema weiter diskutieren möchten, setzen Sie sich einfach mit mir in Verbindung:

Susanne Lorenz *ee08sl@leeds.ac.uk*

Falls Sie mehr über dieses Forschungsprojekt herausfinden möchten, finden Sie mehr Informationen auf dieser Seite (auf Englisch):

http://www.see.leeds.ac.uk/people/s.lorenz

Sie können mir auch auf Twitter folgen:

https://twitter.com/Susanne_Lorenz
