# Supplementary material for: Tailoring the visual communication of climate projections for local adaptation practitioners in Germany and the UK
Source: Philos Trans A Math Phys Eng Sci. 2015 Nov 28;373(2055):20140457. doi: 10.1098/rsta.2014.0457 (PMC4608031; doi:10.1098/rsta.2014.0457)
Supplement: Effects of other sample characteristics on comprehension and use [file rsta20140457supp3.pdf]

Supplementary material for [Lorenz, S., Dessai, S., Forster, P. M. and Paavola, J.], [2015], [Tailoring the visual communication of climate projections for local adaptation practitioners in Germany and the UK], *Phil. Trans. R. Soc. A.* doi: 10.1098/not yet assigned .

Effects of other sample characteristics on comprehension and use

Table 5 Effects of other sample characteristics on assessed comprehension.

				Mann-Whitney U test results			Kruskal-Wallis test results				
				Gender	Educa-tion		Age	Work exper-ience	Project-ions engage-ment	CC Knowledge	Adaptation involve-ment
A	**Pair 1**										
	**ACS - Scatter plot**	**UK**	**U**	1150.5	292.0	χ^2	2.81	7.08	3.16	6.94	6.75
			**z**	-.26	-.50						
		**Ger-many**	**U**	4.65	N/A	χ^2	4.01	4.04	6.52	7.62	1.76
			**z**	-.36	N/A						
	**ACS - Pictograph**	**UK**	**U**	***889.5**	308.5	χ^2	0.23	5.05	3.69	1.81	6.238
			**z**	-2.20	-.20						
		**Ger-many**	**U**	4.28	N/A	χ^2	3.24	3.76	2.92	2.77	2.57
			**z**	-.85	N/A						
	**Pair 2**										
	**ACS - Histogram**	**UK**	**U**	1060.5	308.5	χ^2	6.19	5.74	2.48	3.53	10.99
			**z**	-1.03	-.22						
		**Ger-many**	**U**	.46	N/A	χ^2	6.33	6.10	8.87	2.66	2.50
			**z**	-.34	N/A						
	**ACS – Bubble Plot**	**UK**	**U**	1149.5	209.5	χ^2	2.70	13.47	7.86	4.53	4.52
			**z**	-.25	-1.78						
		**Ger-many**	**U**	.46	N/A	χ^2	2.97	9.48	5.77	6.01	8.40
			**z**	-.36	N/A						

* p < .05 ** p < .01 *** p < .001

Table 6 Effects of other sample characteristics on perceived comprehension, use for self and use for showing to others.

				Chi-square test for independence test results						
				Age	Gender	Educa-tion	Work exper-ience	Projections engagement	CC Know-ledge	Adaptation involve-ment
B	**PC**	**UK**	χ^2	14.64	1.88	1.29	13.77	16.45	13.94	18.36
		**Ger-many**	χ^2	19.67	2.39	N/A	19.21	11.57	8.71	13.94
C	**Use by self**	**UK**	χ^2	16.82	3.86	1.80	39.50	17.43	15.39	14.93
		**Ger-many**	χ^2	17.00	4.24	N/A	31.17	16.25	11.36	19.67
D	**Use for showing to others**	**UK**	χ^2	19.09	2.70	1.98	36.71	15.72	15.69	17.39
		**Ger-many**	χ^2	25.93	4.23	N/A	33.31	25.43	16.49	10.97

Table 5 and 6 summarise the associations between the other sample characteristics and the four criteria (A, B, C and D), the ACSs are broken down for each graph type. With education being a constant in the German sample due to all participants having at least a Bachelor degree, no statistical tests could be undertaken for this variable. The only significant finding can be seen in the UK sample; males (Md = .67, n = 59) have a higher ACS on the pictograph than females (Md = .33, n = 40), U = 889.50, z = -2.20, p = .03, r = .16. There are no further significant effects on assessed or perceived comprehension, use for self and use for showing to others.

Table 7. Effect of Subjective Numeracy Score (SNS) on ACS, PC, Use by self and Use for showing to others as measured by Spearman's Rho

			SNS
ACS	Pair 1		
	Scatter Plot	**UK**	-.04
		**Germany**	.15
	Pictograph	**UK**	**.24***
		**Germany**	.24
	Pair 2		
	Histogram	**UK**	.18
		**Germany**	.09
	Bubble Plot	**UK**	-.03
		**Germany**	**.27***
PC	Pair 1		
	Scatter Plot	**UK**	-.12
		**Germany**	.17
	Pictograph	**UK**	.17
		**Germany**	-.03
	Pair 2		
	Histogram	**UK**	.12
		**Germany**	-.10
	Bubble Plot	**UK**	-.09
		**Germany**	-.09
Use by self	Pair 1		
	Scatter Plot	**UK**	-.13
		**Germany**	-.04
	Pictograph	**UK**	.19
		**Germany**	N/A[1]
	Pair 2		
	Histogram	**UK**	.07
		**Germany**	.15
	Bubble Plot	**UK**	-.01
		**Germany**	.14
Use for showing to others	Pair 1		
	Scatter Plot	**UK**	-.16
		**Germany**	.04
	Pictograph	**UK**	.11
		**Germany**	-.09
	Pair 2		
	Histogram	**UK**	-.03
		**Germany**	.00
	Bubble Plot	**UK**	.15
		**Germany**	-.02

* p < .05 ** p < .01 *** p < .001

We find no systematic influence of subjective numeracy (SNS) on comprehension or use that is consistent across both samples. We see only two significant correlations between SNS and assessed comprehension (ACS) on the Bubble Plot (r = .27, n = 63, p = .03) for the German sample, and between SNS and ACS on the pictograph (r = .24, n = 99, p = .02) for the UK sample. In both instances, higher SNS scores are associated with higher ACS on the respective graph format, but the effect size is small in both cases.

[1] This test cannot be performed as no respondent picked the pictograph for use by self.
